# HIF2α negatively regulates MYCN protein levels and promotes a low-risk noradrenergic phenotype in neuroblastoma

**DOI:** 10.1073/pnas.2516922122

**Published:** 2025-10-21

**Authors:** Juan Yuan, Subhamita Maitra, Eirini Antoniou, Jiacheng Zhu, Wenyu Li, Ilknur Safak Demirel, Kostantinos Toskas, Iria Laura Martinez, Lacin Ozcimen, Henrik Lindehell, Jonas Muhr, Jakob Stenman, Per Kogner, Oscar C. Bedoya-Reina, Susanne Schlisio, Johan Holmberg

**Affiliations:** ^a^Department of Cell and Molecular Biology, Karolinska Institutet, Stockholm 171 77, Sweden; ^b^Department of Molecular Biology, Umeå University, Umeå 901 87, Sweden; ^c^Department of Oncology and Pathology, Karolinska Institutet, Stockholm 171 77, Sweden; ^d^Department of Physiology and Pharmacology, Karolinska Institutet, Stockholm 171 65, Sweden; ^e^Department of Women’s and Children’s Health, Karolinska Institutet, Stockholm 171 65, Sweden; ^f^School of Medical Sciences, University of Örebro, Örebro 701 82, Sweden

**Keywords:** neuroblastoma, HIF2α, MYCN, noradrenergic differentiation, tumor suppression

## Abstract

The role of HIF2α, encoded by *EPAS1*, in neuroblastoma remains controversial. Here, we demonstrate that induction of high levels of HIF2α in MYCN-amplified neuroblastoma cells results in a rapid and profound reduction of the oncoprotein MYCN. This is followed by an upregulation of genes characteristic of noradrenergic cells in the adrenal medulla. Additionally, upon induction of HIF2α, the proliferation rate drops substantially, and cells develop elongated neurite-like protrusions, indicative of differentiation. In vivo HIF2α induction in established xenografts significantly attenuates tumor growth. Notably, analysis of sequenced neuroblastoma patient samples, revealed a negative correlation between *EPAS1* and *MYCN* expression and a strong positive correlation between *EPAS1* expression, high expression levels of noradrenergic markers, and improved patient outcome. This was paralleled by analysis of human developing adrenal medulla datasets wherein *EPAS1* expression was prominent in populations with high expression levels of genes characteristic of noradrenergic chromaffin cells. Our findings show that high levels of HIF2α in neuroblastoma, leads to drastically reduced MYCN protein levels, cell cycle exit, and noradrenergic cell differentiation. Taken together, our results challenge the dogma that HIF2α acts as an oncogene in neuroblastoma.

## Significance

HIF2α has been proposed as a neuroblastoma oncogene and a tractable target for clinical intervention, this has been questioned by several studies. Thus, it is necessary to move beyond correlative studies and better determine the function of HIF2α in neuroblastoma. Our study shows that induced expression of HIF2α in *MYCN-*amplified neuroblastoma substantially reduces MYCN protein levels and attenuates proliferation while it induces several features of noradrenergic differentiation and impedes xenograft tumor growth. Together with bioinformatic analysis of sequenced neuroblastoma patient samples and the developing human adrenal medulla, this couples HIF2α to low-risk neuroblastoma with a substantially better patient outcome and questions whether HIF2α is a neuroblastoma oncogene.

Neuroblastoma arises in the sympathoadrenal lineage and is the most frequent extracranial solid childhood cancer, with a high degree of clinical heterogeneity ranging from spontaneous regression to fatal progression (1). Like in many pediatric malignancies, the frequency of recurring somatic mutations is low. Instead, the disease is characterized by loss and gain of distinct chromosomal regions, for example loss of 1p36, loss of 11q, and gain of 17q. The best characterized neuroblastoma oncogene is *MYCN* which is amplified in roughly 20% of high-risk cases, of which less than 50% survive (2). Even though MYCN has a short protein half-life, the *MYCN* amplification generates extremely high levels of MYCN protein (3). Targeting of MYCN with specific inhibitors has proven challenging, largely due to the intrinsically disorganized structure and lack of a suitable binding pocket. Given the central oncogenic role of MYCN, any process that can significantly reduce MYCN protein levels in an *MYCN*-amplified neuroblastoma would most probably also reduce tumor growth and aggressiveness.

In certain other types of tumors, e.g., subtypes of renal clear cell carcinoma and cases of sporadic paraganglioma, HIF2α is a well-studied oncogenic driver of the disease (4) and a similar role for HIF2α has been suggested in neuroblastoma (5). However, such an oncogenic role for HIF2α has been challenged in several studies (6–10). Despite several hundred bulk RNA-sequenced/arrayed neuroblastoma tumors exhibiting a highly significant correlation between high levels of *EPAS1* expression and low-risk tumors with favorable outcome (7), some studies have continued to argue that HIF2α acts to fuel tumor growth, partly by imposing an immature state with stem cell–like features that blocks differentiation (5, 11–15). However, experimental evidence supporting an oncogenic role for HIF2α in neuroblastoma remains ambiguous.

The generation of neuroblastoma has been tightly linked to the developmental context of the sympathoadrenal system and advances in single-cell sequencing have provided a much more detailed understanding of this lineage. A series of studies has mapped the developmental trajectory of sympathoadrenal cells in both mice and humans (16–23). Importantly, these studies include efforts to align expression profiles of developmental stages with neuroblastoma, which have revealed that neuroblastoma substantially resembles the sympathoblast/neuroblast lineage (16, 20, 21, 23–25). In contrast, cells within the chromaffin lineage exhibit significantly fewer features of high-risk neuroblastoma (20, 21). This dichotomy was substantially underscored in the recently published NBAtlas resource integrating seven previously single cell datasets, wherein analysis suggest that a majority of patient neuroblastoma cells closely resembled neuroblasts rather than cells of the chromaffin lineage (24).

Here, we show that *EPAS1* expression in the developing sympathoadrenal system is correlated with key genes required for noradrenergic chromaffin cell differentiation and function. This reflects previous studies showing that basal levels of HIF2α are required for the normal development of the catecholaminergic phenotype of sympathoadrenal cells, including the expression of enzymes necessary to produce noradrenaline (26). Overexpression of HIF2α in *MYCN*-amplified neuroblastoma cells rapidly induces expression of a cohort of genes also enriched in the chromaffin lineage during normal adrenal gland development, including key enzymes such as *TH, DDC,* and *DBH,* but not *PNMT,* indicating a bias toward noradrenergic chromaffin cells (N-cells) rather than adrenergic E-type cells. This is accompanied by a significant reduction in cellular proliferation. Importantly, levels of the MYCN oncoprotein are rapidly diminished, resulting in decreased expression of MYCN target genes. In addition, induction of HIF2α in already established neuroblastoma xenografts significantly impedes tumor growth. In sum, our data challenge the concept of HIF2α as a neuroblastoma oncogene and show that HIF2α is rather associated with decreased MYCN levels and low-risk tumors as well as noradrenergic chromaffin cell differentiation.

## Results

### EPAS1 Expression Is Positively Correlated With Genes Highly Expressed in Noradrenergic Chromaffin Cells and in Low-Risk Neuroblastoma.

**Fig. 1.**
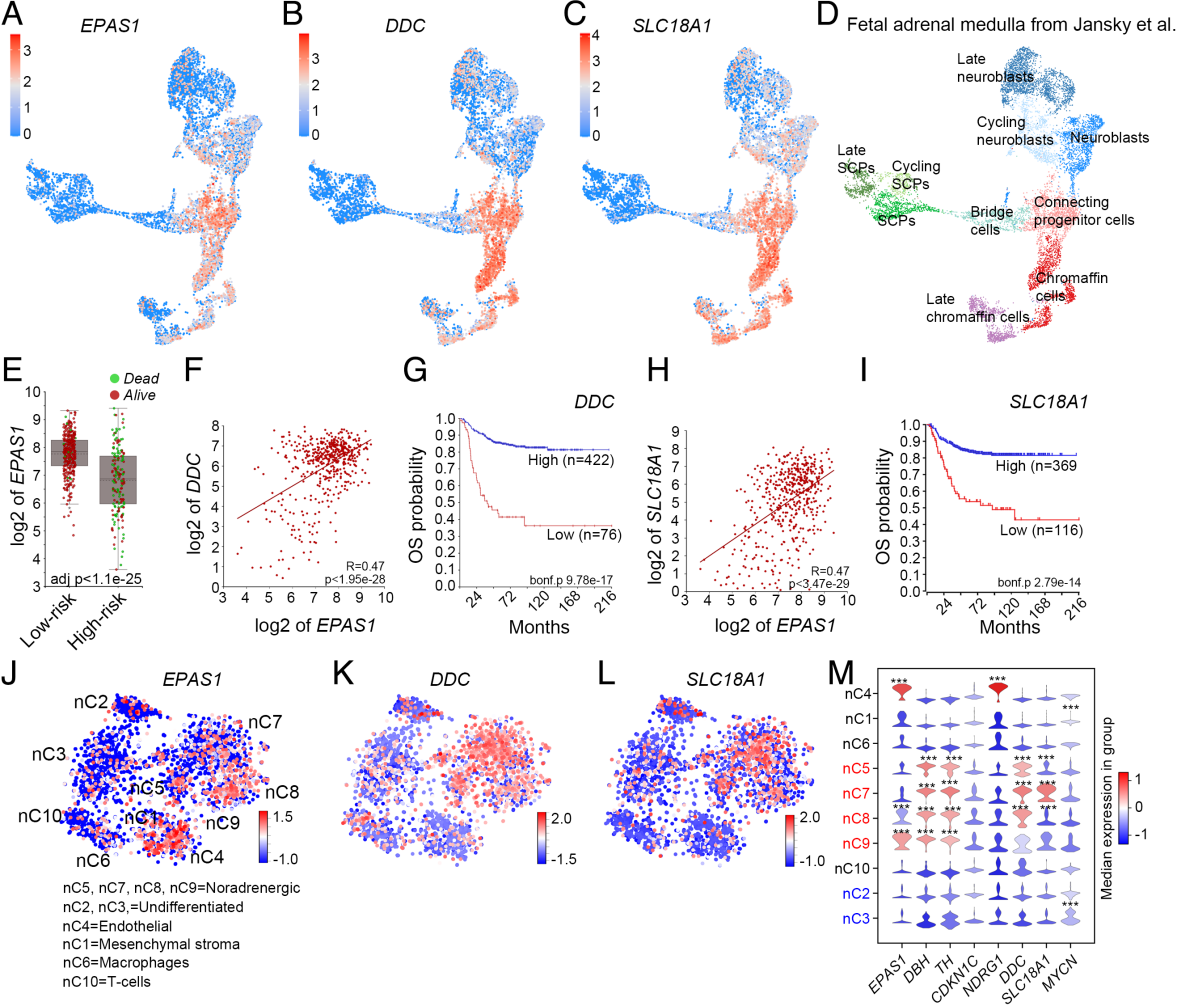
*EPAS1* expression is associated with the chromaffin cell lineage and low-risk neuroblastoma. (*A*–*D*) Visualization of *EPAS1* (*A*), *DDC* (*B*), and *SLC18A1* (*C*) expression in UMAP plot of the developing human adrenal medulla 7 to 17 PCW (*D*), dataset from Jansky et al. (20). (*E*) In neuroblastoma tumors *EPAS1* expression is positively correlated with low-risk tumors lacking *MYCN* amplification. (*F*) Expression of *DDC* and *EPAS1* are positively correlated in neuroblastoma tumors. (*G*) *DDC* expression is correlated to increased overall survival. (*H*) Expression of *SLC18A1* and *EPAS1* are positively correlated in neuroblastoma tumors. (*I*) *SLC18A1* expression is correlated to increased overall survival. (*J*) Mapping of *EPAS1* expression in tSNE of neuroblastoma single cell nuclei show enrichment in endothelial cells, and in noradrenergic low-risk tumor cells, dataset from Bedoya-Reina et al. (16). (*K*) *DDC* expression is enriched for in noradrenergic low-risk tumor cells. (*L*) *SLC18A1* expression is enriched for in noradrenergic low-risk tumor cells. (*M*) Violin plots depicting median expression of the indicated genes in each group. Groups in red font represent noradrenergic neuroblastoma and groups in blue font represent undifferentiated high-risk neuroblastoma. *** Indicates FDR < 0.01.

Through analysis of data from Furlan et al. (18) we previously mapped *EPAS1* expression to the chromaffin lineage during mouse adrenal medulla development (7). To understand whether *EPAS1* expression is associated with the chromaffin lineage also during human development, we utilized previously published data from Jansky et al. (20) (Fig. 1*D*). We plotted the expression of *EPAS1* on to the single cell RNAseq dataset derived from human embryonic adrenal medullas, ranging from 7 to 17 wk post conception (PCW). High levels of *EPAS1* were evident in two populations designated as connecting progenitor cells and as chromaffin cells (Fig. 1*A*). Expression of dopa decarboxylase (*DDC*), an enzyme converting L-DOPA to dopamine, which in turn is a necessary step to produce noradrenaline, is also enriched in these two populations (Fig. 1*B*), as are other key enzymes in this pathway, *TH* and *DBH* (*SI Appendix*, Fig. S1 *A* and *B*). In addition, monoamine transporters *SLC18A1* and *SLC18A2*, necessary for adrenal monoamine homeostasis as well as the cell cycle inhibitor *CDKN1C* and the tumor suppressor *NDRG1*, are also enriched for in the same populations (Fig. 1*C* and *SI Appendix*, Fig. S1 *C* and *D*). There is however no correlation between *EPAS1* and *PNMT*, the enzyme necessary to produce adrenaline from noradrenaline (*SI Appendix*, Fig. S1*E*). This implies that *EPAS1* in human adrenal medulla development is associated with the N-type of chromaffin cells but to a lesser degree with the adrenaline producing E-type chromaffin cells.

Recent studies show that, in neuroblastoma, the majority of cells predominantly resemble sympathoblasts/neuroblasts, rather than chromaffin cells (20, 21, 27). To understand whether *EPAS1* expression also is correlated with chromaffin markers in neuroblastoma tumors, we analyzed the R2 database, utilizing the 498SEQC dataset (28, 29). Expectedly, *EPAS1* expression was significantly higher in the low-risk tumors (Fig. 1*E*). Genes defining the noradrenergic phenotype, for example, key enzymes for the production of monoamines *TH*, *DDC,* and *DBH* as well as the monoamine transporters *SLC18A1* and *SLC18A2* (30) correlate strongly with *EPAS1* expression. In addition, just as for *EPAS1*, high levels of expression of all these genes are strong predictors of improved patient outcome (Fig. 1 *F*–*I* and *SI Appendix*, Fig. S1 *F*–*I* and *K*–*N*). Thus, in combination with previous studies showing that HIF2α is necessary for the catecholaminergic phenotype in sympathoadrenal cells (26) and in the embryonic organ of Zuckerkandl (31), this suggests that high levels of *EPAS1* expression is associated with noradrenergic chromaffin differentiation and that in other cell types of the adrenal medulla the expression levels are generally low. There is a similar lack of overlap between *PNMT* and *EPAS1* expression in neuroblastoma as in the developing adrenal medulla (*SI Appendix*, Fig. S1*J*), potentially reflecting a N-type like, rather than E-type like, chromaffin identity in cells with elevated *EPAS1* levels.

Through analysis of several bulk sequenced datasets of neuroblastoma patient samples we have previously shown that high expression levels of *EPAS1* are strongly correlated with increased patient survival and features typical of low-risk tumors (6, 7, 32). To understand whether this also could be reflected in single cell sequenced neuroblastoma, we inspected 11 previously single cell sequenced tumors (16). This revealed that *EPAS1*, besides from a strong enrichment in endothelial cells, is significantly enriched in clusters nC8 and nC9 from noradrenergic low-risk tumors (NOR-clusters) characterized by high expression of noradrenergic markers such as *DDC* and *SLC18A1* and significantly negatively correlated with undifferentiated high-risk neuroblastoma with high *MYCN* expression (Fig. 1 *J*–*M* and *SI Appendix*, Fig. S1 *O*–*S*) (16). Thus, *EPAS1* is associated with a more differentiated noradrenergic cellular state. In contrast, neuroblastoma cells belonging to more undifferentiated high-risk tumors exhibit lower levels of *EPAS1* (Fig. 1 *J* and *M*). However, in two NOR clusters (nC6 and nC7) there was no significant enrichment of *EPAS1* and in one undifferentiated cluster (nC2) there was no significant enrichment of *MYCN*, so to further validate that *EPAS1* expression is associated with a more differentiated cellular state, we utilized a recently published study wherein single cell sequencing data from 6 different studies composed of 126 872 cells from 68 samples from 61 different neuroblastoma patients had been combined (24). This resource is available in the R2 database (28). Eight clusters were defined by differentially expressed genes and gene set enrichment analysis (GSEA). These clusters also correlated to high-risk vs low/intermediate-risk, with cluster “c0. Differentiated” incorporating cells from predominantly low/intermediate-risk tumors (*SI Appendix*, Fig. S2 *A* and *B*). We mapped the expression of *EPAS1*, *DDC, DBH,* and *MYCN* in the eight clusters (*SI Appendix*, Fig. S2 *C*–*K*). Supporting our previous analysis, *EPAS1*, *DDC, and DBH* expression is enriched for in the “c0. Differentiated” cluster but depleted in the “c6. MYCN” cluster whereas the *MYCN* transcript was strongly enriched for in the “c6. MYCN” dominated by high-risk tumor cells (*SI Appendix*, Fig. S2 *C*–*K* and Dataset S1). Furthermore, high *EPAS1* expression levels correlated significantly with both *DDC* and *DBH* expression, whereas it was negatively correlated with *MYCN* expression (*SI Appendix*, Fig. S2 *K*–*M*).

To map the expression of *EPAS1* in neuroblastoma *in situ,* we performed RNA-scope with probes for *EPAS1*, *TH,* and the endothelial marker *ENG* (*Endoglin*) in tumors of different stages. In low-risk stage 4S and 2B tumors *EPAS1* colocalized with *ENG* but there were also several areas with strong colocalization between *EPAS1* and *TH* where *ENG* was not present (*SI Appendix*, Fig. S3 *A* and *B*). In a high-risk stage 4 tumor, *EPAS1* was typically only expressed in blood vessels expressing high levels of *ENG.* Also, in a restricted area of the tumor containing strong focal expression of *TH EPAS1* expression was primarily restricted to *ENG^+^* cells, indicating that in high-risk tumors *EPAS1* is predominantly expressed in stromal endothelial cells. (*SI Appendix*, Fig. S3*C*).

### High Levels of HIF2α Promote Reduced Proliferation and Features of Noradrenergic Chromaffin Differentiation in MYCN-Amplified Neuroblastoma Cells.

**Fig. 2.**
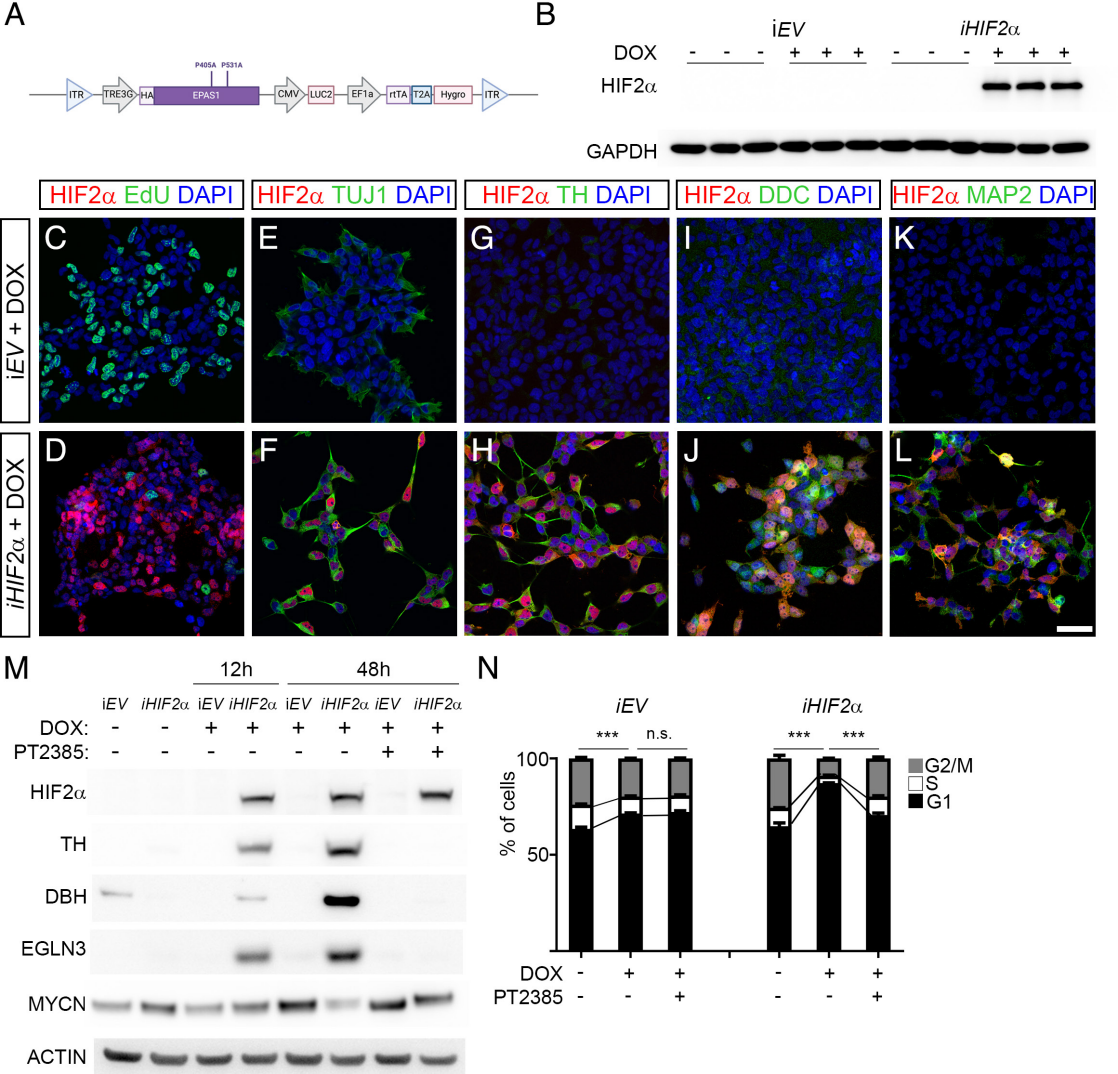
Overexpression of HIF2α in *MYCN*-amplified LAN-1 neuroblastoma cells leads to reduced proliferation and upregulation of chromaffin associated factors. (*A*) Schematic representation of *piggyBac* construct for doxycycline inducible expression of a stabilized form of *EPAS1* (P450A, P531A). (*B*) Doxycycline induced expression of *EPAS1* (*iHIF2α)* in LAN-1 neuroblastoma cells. (*C* and *D*) Immunostaining with HIF2α (red) combined with EdU (green) and DAPI (blue) in *iEV* control cells (*C*) and in *iHIF2α* (*D*) cells treated with doxycycline. (*E* and *F*) Immunostaining with HIF2α (red) combined with TUJ1 (green) and DAPI (blue) in *iEV* control cells (*E*) and in *iHIF2α* (*F*) cells treated with doxycycline. (*G* and *H*) Immunostaining with HIF2α (red) combined with TH (green) and DAPI (blue) in *iEV* control cells (*G*) and in *iHIF2α* (*H*) cells treated with doxycycline. (*I* and *J*) Immunostaining with HIF2α (red) combined with DDC (green) and DAPI (blue) in *iEV* control cells (*I*) and in *iHIF2α* (*J*) cells treated with doxycycline. (*K* and *L*) Immunostaining with HIF2α (red) combined with DDC (green) and DAPI (blue) in *iEV* control cells (*K*) and in *iHIF2α* (*L*) cells treated with doxycycline. (*M*) Western blot showing upregulation of HIF2α, TH, DBH, and EGLN3 12 h after doxycycline induction. Addition of the HIF2α inhibitor PT2385 at 48 h reverses increase of TH, DBH, and EGLN3, but MYCN protein levels are restored. ACTIN is shown as loading control. (*N*) Cell cycle analysis after PI-staining shows a decrease in cells in S-phase and G2/M-phase. This decrease is reversed upon treatment with PT2385 in *iHIF2α* cells. Cell cycle data are represented as mean ± SD; *P*-value of differences in S-phases was calculated with ANOVA with Tukey’s multiple comparisons test. (Scale bar, L = 50μm.)

The clear correlation of *EPAS1* with increased patient survival, low-risk tumors, and noradrenergic differentiation prompted us to test whether increased levels of HIF2α in neuroblastoma cells would affect key cellular properties such as proliferation and differentiation. To address this, we utilized the *piggyBac* transposon system (33) to overexpress a stabilized version of HIF2α (34) under the control of doxycycline (*iHIF2α*) (Fig. 2 *A* and *B*). In *MYCN*-amplified LAN-1 neuroblastoma cells, induction of *iHIF2α* results in slowed cellular growth and reduced incorporation of 5-ethynyl-2′-deoxyuridine (EdU) (Fig. 2*C*, *SI Appendix*, Fig. S4 *A* and *B*). Immunostaining with antibodies specific for TUJ1, TH, and DDC 48 h after induction, revealed changes in morphology with *iHIF2α* expressing cells extending TUJ1^+^ neurite-like protrusions (Fig. 2 *D*–*E* and *SI Appendix*, Fig. S4 *C* and *D*) and substantially increased levels of TH, DDC, and MAP2 (Fig. 2 *F*–*L* and *SI Appendix*, Fig. S4 *E*–*J*).

To further validate that the induction of TH and DBH as well as the reduced proliferation was a response to high HIF2α activity, we used the HIF2α inhibitor PT2385 which inhibits the dimerization of HIF2α with ARNT1 and prevents activation of HIF2α target genes (35–37). Already after 12 h of doxycycline treatment there was an increase in protein levels of TH, DBH, and the HIF2α target EGLN3. After 48 h this effect was even more pronounced. However, treatment with PT2385 abolished the effect without affecting induced HIF2α levels (Fig. 2*M*). We could also detect a reduction in MYCN protein levels upon *iHIF2α* induction, this decrease was partially reversed by PT2385 (Fig. 2*M*). To quantify the effects on cellular proliferation we performed propidium iodide (PI) staining. This revealed a significant reduction in cells in S and G2/M phase upon *iHIF2α* induction which was reversed upon PT2385 treatment (Fig. 2*N*).

We also compared the HIF2α protein levels between doxycycline induced *iHIF2α* LAN-1 cells and human endothelial cells (HUVEC) and in VHL deficient clear cell renal cell carcinoma (ccRCC) cells (786-O) (*SI Appendix*, Fig. S4*K*). This indicated higher levels of HIF2α in LAN-1 cells upon doxycycline induction than present in these cell lines which have been reported to contain robust levels of HIF2α protein. To further ensure that the effects of *iHIF2α* were not merely consequences of random transcription factor overexpression, we proceeded to overexpress *HIF1α, ASCL1* and *PHOX2B* in LAN-1 and Kelly cells. Neither transcription factor resulted in decreased levels of MYCN (*SI Appendix*, Fig. S5 *A*–*C*). *HIF1α* overexpression did not significantly alter the TH levels in neither LAN-1 nor Kelly cells (*SI Appendix*, Fig. S5*A*). However, upon *PHOX2B* overexpression in LAN-1 cells there was a reduction of TH protein levels (*SI Appendix*, Fig. S5*C*). We performed TUJ1 staining in LAN-1 cells to visualize any changes in morphology upon overexpression of the same transcription factors. However, no such morphological changes were evident (*SI Appendix*, Fig. S5 *D*, *G*, *J*, and *M*). Immunostaining for TH revealed that in a restricted number of cells overexpressing *ASCL1* there was increase of TH immunoreactivity, none of the other transcription factors had this effect (*SI Appendix*, Fig. S5 *E*, *H*, *K*, and *N*). MYCN immunostaining showed no decrease in MYCN protein levels in cells overexpressing any of the transcription factors (*SI Appendix*, Fig. S5 *F*, *I*, *L*, and *O*).

### Overexpression of EPAS1/HIF2α in MYCN-Amplified Neuroblastoma Cells Induces a Transcriptional Response Characterized by Genes Highly Expressed in Noradrenergic Cells of the Adrenal Medulla.

**Fig. 3.**
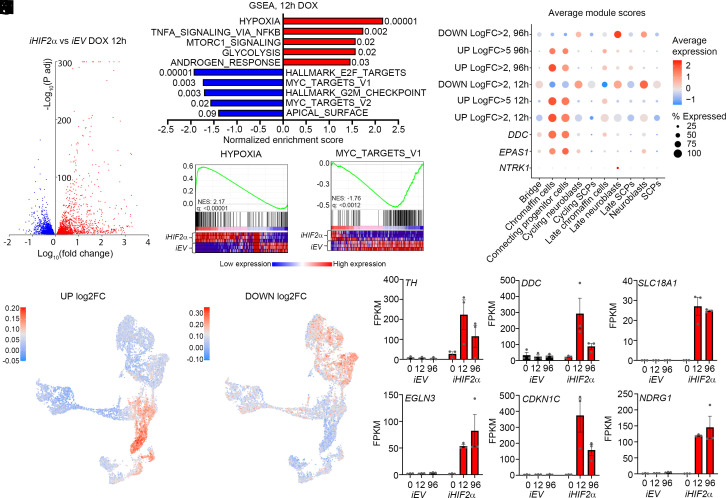
Overexpression of *EPAS1* rapidly induces expression of genes associated with hypoxia and the chromaffin cell lineage while there is a reduction of MYCN targets and genes associated with cell cycle progression. (*A*) Volcano plot showing genes upregulated (red) and downregulated (blue) 12 h after induction of *iHIF2α*. (*B*) Gene set enrichment analysis (GSEA) of the genes from (*A*), numbers indicate FDR. (*C*) GSEA of the “HYPOXIA” gene set. (*D*) GSEA of the “MYC_TARGETS_V1.” (*E*) Dot plot showing the average gene module scores for the indicated genes and gene groups. (*F*) Genes upregulated >Log2FC in (*A*) plotted on the developing adrenal medulla from Jansky et al. (*G*) Genes downregulated >Log2FC in (*A*) plotted on the developing adrenal medulla from Jansky et al. (*H*–*M*) Expression of the indicated genes *iEV* and *iHIF2α* at 0, 12, and 96 h after doxycycline treatment.

To investigate the full transcriptional response to HIF2α, we prepared cDNA libraries of three different clones expressing *iHIF2α* for sequencing 12 h and 96 h after doxycycline induction. Analysis showed that at 12 h 2,801 genes were significantly upregulated (adj. *P* < 0.05) and 2,652 genes were downregulated (Fig. 3*A* and Datasets S2 and S3). At 96 h 1,828 genes were upregulated and 1,193 were downregulated (*SI Appendix*, Fig. S4*A* and Datasets S4 and S5). GSEA utilizing the “Hallmarks” collection of gene sets from Broad Institute (38, 39), revealed that both at 12 h and 96 h induction of HIF2α resulted in the upregulation of several gene sets including the “HYPOXIA” gene set (Fig. 3 *B* and *C* and *SI Appendix*, Fig. S6 *B* and *C*). In the *iHIF2α* induced cells there was a strong negative correlation with terms associated with MYC-targets and proliferation (Fig. 3 *B* and *D* and *SI Appendix*, Fig. S6 *B* and *D*). The strong negative correlation with cell cycle related gene sets reflects the reduction in cells in S-Phase (Fig. 2*N*) and was further underscored when cell numbers were counted over 4 four days, whereupon *iHIF2α* induced LAN-1 cells exhibited a significant reduction in growth compared to *iEV* cells (*SI Appendix*, Fig. S6*E*).

To understand whether the upregulated or downregulated gene signatures resemble any specific cellular populations in the developing human adrenal medulla, we utilized the Jansky et al. (20) dataset to compute gene module scores of up- and downregulated genes. Both at 12 h and 96 h genes upregulated log2foldchange (log2fc) >2 and >5 were enriched in the “Chromaffin cells” and “Connecting progenitor cells” cell types (Fig. 3 *E* and *F* and *SI Appendix*, Fig. S6*F*). In contrast, genes downregulated log2fc >2 were enriched for in other cell types, in particular in “Neuroblasts” and “Late neuroblasts” (Fig. 3 *E* and *G* and *SI Appendix*, Fig. S6*G*). Downregulated genes at both timepoints were depleted in “Chromaffin cells” and “Connecting progenitor cells” (Fig. 3*E*). To understand the effect of elevated HIF2α levels on the expression of specific noradrenergic chromaffin associated genes, over time, we plotted the FPKM of *TH, DDC,* and *SLC18A1* which all showed robust increased expression levels upon *iHIF2α* induction (Fig. 3 *H*–*J*). In addition, we plotted expression levels of HIF2α target gene *EGLN3*, the cell cycle inhibitor *CDKN1C* and the tumor suppressor *NDRG1* which is a direct negative target of MYCN regulation (40) (Fig. 3 *K* and *M*). There was however no increase in *PNMT* expression.

A recent study from Bishop and colleagues (41) shows how high levels of HIF2α in the mouse adrenal medulla promotes a *Pnmt1^-^/Epas1^+^/Rgs5^+^* noradrenergic phenotype that produces noradrenaline in contrast to the *Pnmt1^+^/Epas1^-^/Rgs5^-^* adrenaline producing cells. In our system the induction of *iHIF2α* induced high levels of *RGS5* (up ~6-fold, adj. *P* < 1.3e−44) at 96 h as well as the atypical mitochondrial regulators *COX4I2* (up~259-fold, adj. *P* < 8.7e−09) at 12 h and *NDUFA4L2* (up~5.fold, adj. *P* < 1.2e−04) at 96 h, which also were upregulated in the mouse adrenal medulla upon induction of HIF2α (41). In addition, analysis of the 498SEQC dataset showed that *RGS5, NDUFA4L2,* and *COX4I2* are all significantly correlated with *EPAS1* expression in neuroblastoma (*SI Appendix*, Fig. S6 *H*–*J*). Both *RGS5* and *NDUFA4L2* are profoundly correlated with increased overall survival (*SI Appendix*, Fig. S6 *K* and *L*), whereas *COX4I2* exhibited no significant correlation with overall survival (*SI Appendix*, Fig. S6*M*).

### Induction of EPAS1/HIF2α in MYCN-Amplified Neuroblastoma Cells Rapidly Depletes MYCN Protein Levels, Followed by an Increase in Enzymes Necessary for Noradrenaline Synthesis.

**Fig. 4.**
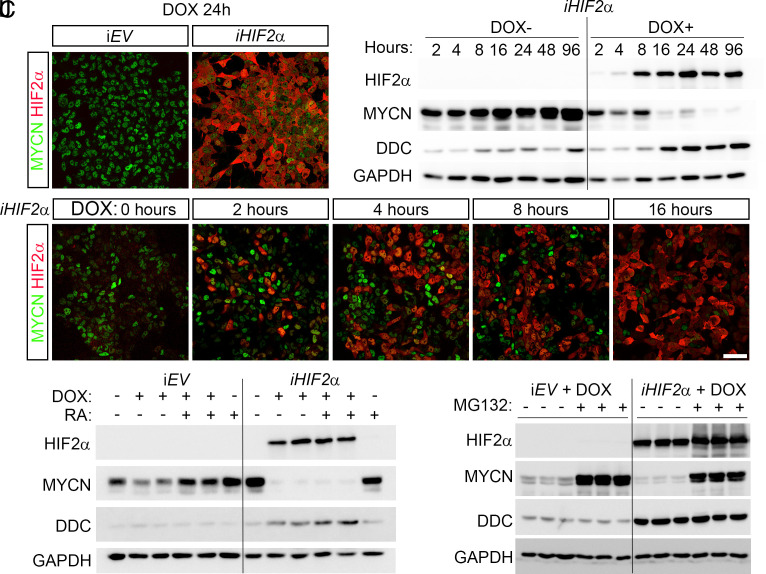
Induction of *iHIF2α* depletes MYCN protein levels prior to the increase of DDC. (*A* and *B*) In *iEV* expressing cells there is no induction of HIF2α and MYCN protein levels remain high, 24 h after doxycycline treatment (*A*), whereas in *iHIF2α* cells there is an upregulation of HIF2α and a reduction in MYCN protein levels (*B*). (*C*) Western blot showing protein levels of HIF2α, MYCN, DDC, and GAPDH at the indicated time points after doxycycline induction in *iHIF2α* cells. (*D*–*H*) Immunostaining with antibodies for MYCN (green) and HIF2α (red) at the indicated time points after doxycycline induction. (*I*) Western blot showing the indicated protein levels in LAN-1 cells treated with retinoic acid (RA) with or without doxycycline induction. (*J*) Western blot showing the indicated protein levels in LAN-1 cells treated with the proteasome inhibitor MG132 after 24 h of doxycycline induction. [Scale bar in (*H*) = 50 μm.]

Despite a significant negative correlation between *MYCN* and *EPAS1* expression in 498 sequenced clinical samples (6) and a HIF2α dependent reduction in MYCN protein levels (Fig. 2*M*), we observed no significant decrease in *MYCN* expression upon *iHIF2α* induction. The observed downregulation of MYC targets upon *iHIF2α* induction prompted us to investigate how elevated levels of HIF2α affects MYCN protein levels. Following 24 h of doxycycline treatment there was a clear reduction in MYCN immunoreactivity (Fig. 4 *A* and *B* and *SI Appendix*, Fig. S7 *A*–*D*). To better map the consequences for MYCN over time we conducted a time-course analysis over 2 to 96 h postdoxycycline induction. This revealed that rising levels of HIF2α resulted in reduced MYCN protein levels, and despite a brief paucity in this reduction at 8 h, the levels had at 16 h dropped substantially, concurrent with an increase in DDC levels (Fig. 4*C*). Immunostaining at short intervals revealed that there was a window at 2 h when MYCN and HIF2α proteins were detectable in the same cells. However, already after 4 to 8 h, there was a distinct loss of MYCN as HIF2α levels rose (Fig. 4 *C*–*G* and *SI Appendix*, Fig. S7 *E*–*N*). The reduction in MYCN protein levels aligns with the gene sets enriched for downregulated genes as indicated by GSEA. Retinoic acid (RA) is used as an adjuvant treatment in patients with high-risk tumors and has a strong differentiation inducing effect in certain neuroblastoma cell lines, however not in LAN-1 cells. In LAN-1 cells the response to *iHIF2α* induction was a depletion of MYCN protein levels and induction of DDC, whereas RA alone failed to promote any such response (Fig. 4*I*). The combined induction of *iHIF2α* and RA treatment appears to increase the levels of DDC even further, suggesting that high levels of HIF2α might restore RA sensitivity in LAN-1 cells. Treatment with the proteasome inhibitor MG132 restored MYCN protein levels in *iHIF2α* cells treated with doxycycline for 24 h (Fig. 4*J*). Thus, high levels of HIF2α results in targeting MYCN for proteasomal degradation and even in the presence of MG132 the levels of MYCN were lower than in control cells (*iEV* + DOX). In contrast, the levels of DDC induced by *iHIF2α* expression, were unaffected by MG132 treatment, indicating that HIF2α induces expression of the *DDC* gene rather than influencing its protein stability (Fig. 4*J*).

To validate the effects of high levels of HIF2α in another *MYCN*-amplified neuroblastoma cell line we established a similar *piggyBac* system in the SK-N-BE(2) neuroblastoma cells. As in the LAN-1 cells doxycycline induced high levels of HIF2α and reduced incorporation of EdU (*SI Appendix*, Fig. S8 *A* and *B*). Immunostaining for TUJ1, TH, DDC, and MAP2 revealed increased immunoreactivity and changes in cellular morphology similar to the changes in LAN-1 cells (*SI Appendix*, Fig. S8 *C*–*J*). As in the LAN-1 cells *iHIF2α* induction resulted in decreased MYCN immunoreactivity (*SI Appendix*, Fig. S8 *K*–*L*). We also investigated whether there was cell autonomous loss of MYCN in the cells that upregulate HIF2α. Reflecting the effect in LAN-1 cells, immunostaining for MYCN and HIF2α revealed an inverse correlation in protein levels (*SI Appendix*, Fig. S8 *M*–*Q*). This pattern was also supported by western blot analysis reflecting the time-dependent decrease in MYCN protein levels and an increase in DDC, TH, and DBH protein levels (*SI Appendix*, Fig. S8*R*). Notably, the brief paucity of MYCN reduction in the LAN1 cells at 8 h after *iHIF2α* induction was also evident in the SK-N-BE(2) cells (S4R). PI staining showed a significant reduction in the number of cells in S phase upon induction of *iHIF2α* (*SI Appendix*, Fig. S8*S*). A significant reduction in cellular growth over four days was also detectable (*SI Appendix*, Fig. S8*T*).

We transiently overexpressed *HIF2α* in the *MYCN*-amplified Kelly neuroblastoma cells. Also, in these cells this resulted in a reduction of MYCN protein levels and in increase in TH protein levels (*SI Appendix*, Fig. S8*U*).

### Induction of EPAS1/HIF2α in Non-MYCN-Amplified Neuroblastoma Cells Induces a Transcriptional Response Characterized by Hypoxia and Reduced MYC-Target Gene Expression.

**Fig. 5.**
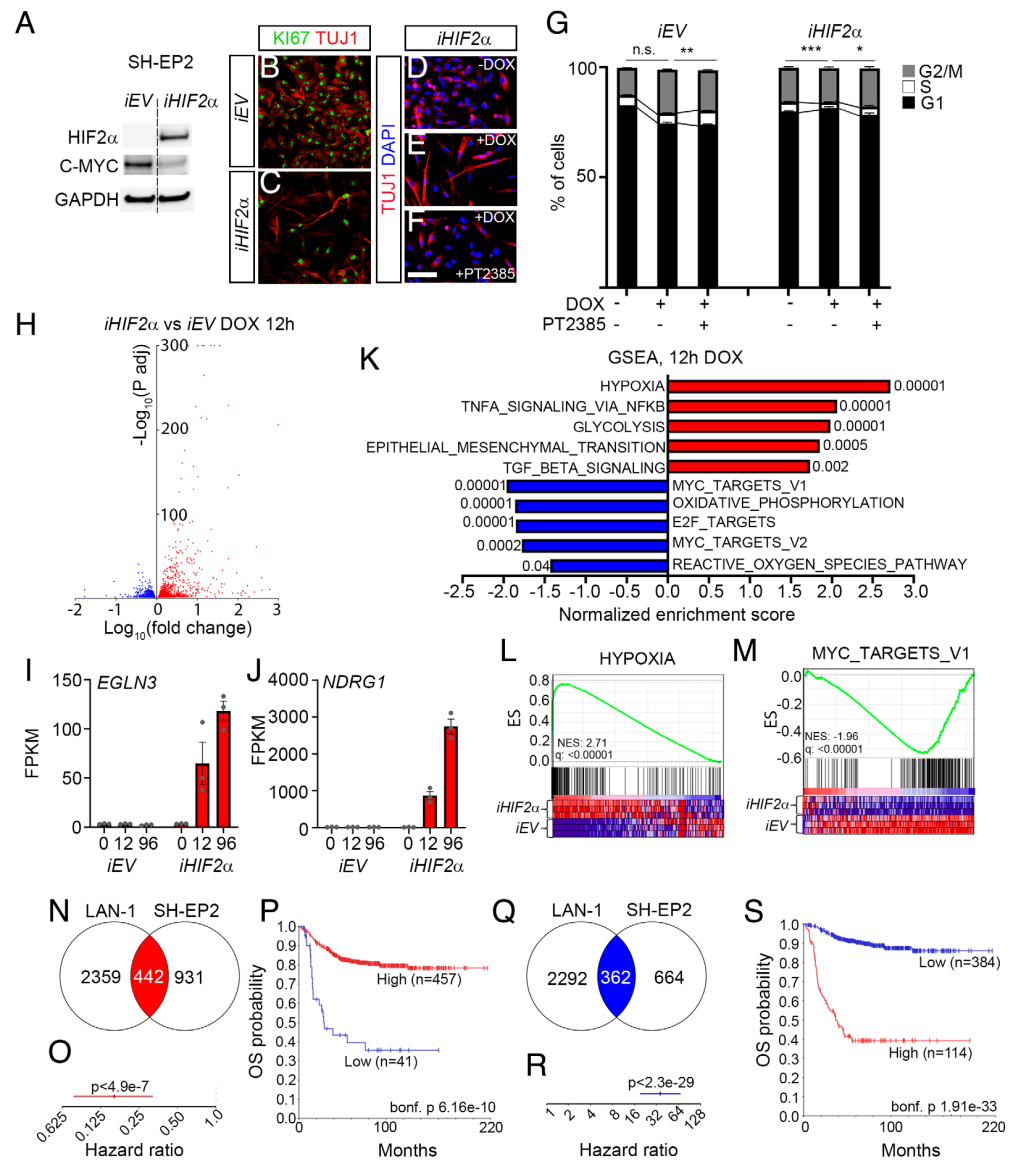
Induction of *EPAS1*/HIF2α in non-*MYCN*-amplified neuroblastoma cells induces a transcriptional response characterized by hypoxia and reduced MYC-target gene expression. (*A*) Western blot showing reduction in MYC upon overexpression of *iHIF2α* in SH-EP2 neuroblastoma cells. GAPDH is shown as loading control. (*B* and *C*) Immunostaining with TUJ1 (red) combined with KI67 (green) in *iEV* control cells (*B*) and in *iHIF2α* (*C*) cells treated with doxycycline. (*D*–*F*) Immunostaining with TUJ1 (red) combined with DAPI (blue) in *iHIF2α* SH-EP2 cells without doxycycline (*D*), with doxycycline (*E*) and with doxycycline and PT2385 (*F*). (*G*) Cell cycle analysis after PI-staining shows a decrease in cells in S-phase and G2/M-phase. This decrease in S-phase is reversed upon treatment with PT2385 in *iHIF2α* cells. (*H*) Volcano plot showing genes upregulated (red) and downregulated (blue) 12 h after induction of *iHIF2α*. (*I* and *J*) Expression levels of *EGLN3* (*I*) and *NDRG1* (*J*) 0, 12, and 96 h after doxycycline induction. (*K*) GSEA of the genes from (*A*), numbers indicate FDR. (*L*) GSEA of the “HYPOXIA” gene set. (*M*) GSEA of the “MYC_TARGETS_V1” gene set. (*N*) Genes commonly upregulated in *iHIF2α* LAN-1 and SH-EP2 cells, 12 h after doxycycline induction. (*O*) Hazard ratio of commonly upregulated genes in (*N*), in the 498SEQC neuroblastoma cohort. (*P*) Expression of commonly upregulated genes in the 498SEQ neuroblastoma cohort is correlated with increased overall survival. (*Q*) Genes commonly downregulated in *iHIF2α* LAN-1 and SH-EP2 cells, 12 h after doxycycline induction. (*R*) Hazard ratio of commonly downregulated genes in (*Q*), in the 498SEQC neuroblastoma cohort. (*S*) Expression of commonly downregulated genes in the 498SEQ neuroblastoma cohort is correlated with decreased overall survival. (Scale bar in *F* = 50 μm.)

To investigate whether *iHIF2α* induction also could provoke a response in neuroblastoma cells lacking *MYCN*-amplification we generated three clones with the same doxycycline inducible *iHIF2α* construct in the SH-EP2 neuroblastoma cell line. SH-EP2 cells are a subclone from SK-N-SH cells and exhibit a distinct mesenchymal phenotype. We could not detect any expression of TH upon induction of *iHIF2α*, presumably due to the mesenchymal cellular state of SH-EP2 cells. Endogenous levels of MYCN in SH-EP2 cells are not detectable, however there was a reduction in MYC protein levels (Fig. 5*A*). Immunostaining for TUJ1 and KiI67 after *iHIF2α* induction revealed a change in cellular morphology to a more elongated morphology and with less Ki67 (Fig. 5 *B* and *C*). The change in morphology was reversed upon treatment with the HIF2α inhibitor PT2385 (Fig. 5 *D*–*F*). The reduction in Ki67 immunoreactivity implies a reduction in cellular proliferation. Hence, we performed PI-staining to reveal the effects of *iHIF2α* induction. Upon *iHIF2α* induction there was a significant decrease in the number of cells in S- and G2/M-phase, partly reversible by PT2385 (Fig. 5*G*). To investigate potential differences and similarities in the transcriptional response to *iHIF2α* induction between the MYCN amplified LAN-1 cells and the mesenchymal non-MYCN amplified SH-EP2 cells we performed RNA-sequencing of the three *iHIF2α* SH-EP2 clones at 12 and 96 h after doxycycline treatment (Fig. 5*H* and *SI Appendix*, Fig. S9*A*). This resulted in the upregulation of 1,373 genes at 12 h and 2,731 genes at 96 h, 1,025 genes were downregulated at 12 h and 2,611 genes downregulated at 96 h (Datasets S5–S9). The endogenous expression levels of *DBH, DDC, SLC18A1,* and *TH* range between 0-1.5 FPKM reflecting the mesenchymal phenotype of SH-EP2 cells and we could not detect any significant increase upon *iHIF2α* induction (*SI Appendix*, Fig. S9 *B*–*E*). In contrast, both the HIF2α target gene *EGLN3* and the MYC suppressed gene *NDRG1* exhibited a substantial increase, with *NDRG1* increasing more than 170 times (Fig. 5 *I* and *J*). GSEA revealed a strong enrichment of the HYPOXIA gene category for the upregulated genes (Fig. 5 *K*–*L* and *SI Appendix*, Fig. S9 *F* and *G*). In contrast, downregulated genes generated a strong enrichment of cell cycle linked gene categories and notably MYC-TARGETS V1 and V2 (Fig. 5 *K* and *M* and *SI Appendix*, Fig. S9 *G* and *H*).

To investigate whether genes commonly up- and downregulated in LAN-1 and SH-EP2 cells (Fig. 5 *N* and *Q* and *SI Appendix*, Fig. S9 *I* and *L*) could predict clinical outcome we used 498SEQC dataset from the R2 database to generate hazard ratios (HR) and Kaplan–Meier curves for overall survival. This analysis revealed HR values of commonly upregulated genes generated HR values of 0.16 at 12 h (Fig. 5*O*) and 0.20 at 96 h (*SI Appendix*, Fig. S9*J*). In contrast, commonly downregulated genes had hazard ratios of 36 at 12 h (Fig. 5*R*) and 32 at 96 h (*SI Appendix*, Fig. S9*M*). The corresponding overall survival at the different time-points revealed a strong relationship between genes commonly upregulated by *iHIF2α* and improved overall survival (Fig. 5*P* and *SI Appendix*, Fig. S9*K*). Commonly downregulated genes were significantly correlated with decreased overall survival (Fig. 5*S* and *SI Appendix*, Fig. S9*N*).

The SH-SY5Y cells is a more noradrenergic clone of the SK-N-SH. Transient overexpression of *HIF2α* in SH-SY5Y cells resulted in a reduction in MYC levels and an increase in TH and DBH levels (*SI Appendix*, Fig. S9*O*).

In both LAN-1 and SH-EP2 cells there was an enrichment for the “TNFA_signaling_VIA_NFKB” gene set, but the *TNF*α gene itself was not upregulated. However, upon comparison of the 200 genes of this category with genes upregulated 12 and 96 h after induction of *iHIF2α*, we identified 35 common genes (*SI Appendix*, Fig. S10*A*). To understand whether these 35 common genes were predictive of clinical outcome we utilized the 498SEQC cohort in the R2 database. This revealed that 23 of these genes were significantly correlated with increased overall survival whereas only two genes were associated with decreased overall survival. Notably, these two genes also had the least significant *P*-value (*SI Appendix*, Fig. S10*B*). The gene with the most significant correlation with overall survival was NFKB2 (*SI Appendix*, Fig. S10 *B* and *C*) which also is positively correlated with *EPAS1* expression and negatively correlated with *MYCN* (*SI Appendix*, Fig. S10 *D* and *E*) in the 498SEQC cohort and increased more than fivefold 96 h after *iHIF2α* induction (*SI Appendix*, Fig. S10*F*). Analysis in SH-EP2 cells revealed a similar pattern with 53 commonly upregulated genes out of which 28 predicted increased overall survival and 4 predicted decreased survival (*SI Appendix*, Fig. S10 *G* and *H*).

### In Neuroblastoma Xenografts, Induction of EPAS1/HIF2αA Impedes Tumor Growth and Triggers Expression of Genes Highly Expressed in Noradrenergic Chromaffin Cells.

**Fig. 6.**
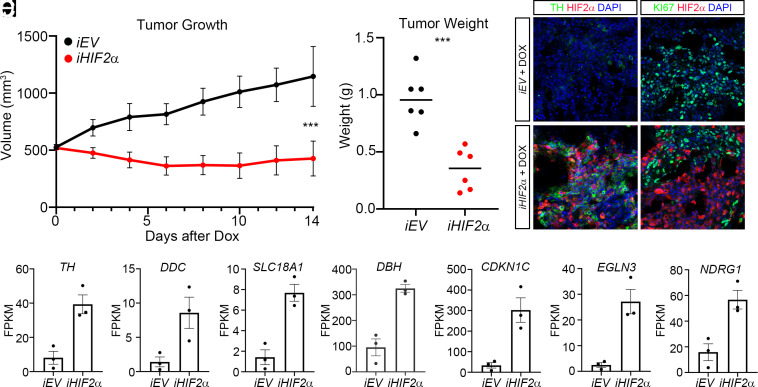
Induction of *iHIF2α* in formed xenograft tumors impedes tumor growth and upregulates expression of chromaffin genes. (*A*) Induction of *iHIF2α* in xenograft tumors with an average volume of 500 mm^3^ significantly impedes tumor growth. (*B*) Tumor weight at the endpoint for doxycycline treated *iEV* tumors (black) and *iHIF2α* tumors (red). (*C* and *D*) Immunostaining for TH (green) and HIF2α (red) in doxycycline treated xenografted tumors, *iEV* (*C*) and *iHIF2α* (*D*). (*E* and *F*) Immunostaining for KI67 (green) and HIF2α (red) in doxycycline treated xenografted tumors, *iEV* (*E*) and *iHIF2α* (*F*). (*G*–*M*) Expression of the indicated genes in doxycycline treated xenografts. Tumor growth in *A* is represented as mean tumor volume ± SD, **P < 0.01, two-way ANOVA, (n = 6 per group), with Šídák’s multiple comparisons test. In *B*, ***P < 0.001, unpaired *t* test with Welch’s correction. (Scale bar in *F* = 50 μm.)

The strong reduction of MYCN protein levels upon activation of the HIF2α pathway in *MYCN-*amplified cells coupled to the drop in proliferation and expression of noradrenergic genes (Fig. 4) indicate that rather than acting as a neuroblastoma oncogene, HIF2α has potential capacity to attenuate tumor growth. To test this in an animal model we xenografted nude mice with LAN-1 cells expressing *iHIF2α* or the *iEV* empty vector as a control. Tumors were allowed to grow to 500 mm^3^ before treatment with doxycycline was initiated. Although a proportion of cells did not respond to doxycycline, the induction of HIF2α efficiently impeded tumor growth (Fig. 6 *A*-*B*). Neuroblastoma cells in the HIF2α-induced tumors, exhibiting high levels of HIF2α also have high levels of TH, but low levels of Ki67, whereas control tumors exhibit high levels of Ki67 and low levels of TH (Fig. 6 *C*-*F*).

Despite a heterogenous cellular response to doxycycline-mediated induction of *iHIF2a* (Fig. 6 *D* and *F*) we performed gene expression analysis on retrieved post treated tumors. Besides high levels of *EPAS1* (Fig. 6*G*), a similar cohort of genes to those upregulated after 12 h and 96 h after *in vitr*o induction (Fig. 3 *H*–*M*) (*TH, DDC, SLC18A1, DBH, CDKN1C, EGLN3,* and *NDRG1*) was upregulated in the xenografts wherein *iHIF2α* was induced (Fig. 6 *H*–*O*). Thus, *EPAS1* correlated well with expression of markers of noradrenergic cells within the adrenal medulla, e.g. *DBH* or *TH* as well as the cell cycle inhibitor *CDKN1C* and the tumor suppressor *NDRG1* (Fig. 6 *G*–*O*). There was however no increase in *PNMT* expression. This supports our previous analysis of *EPAS1* expression within the same lineage during mouse development (7) as well as our analysis of *EPAS1* expression within the development of the human lineage and in neuroblastoma (Fig. 1 and *SI Appendix*, Fig. S1).

## Discussion

Our study reveals that high levels of *EPAS1* are strongly associated with genes highly expressed in noradrenergic cells of the adrenal medulla. This gene repertoire is also predictive of low-risk tumors lacking *MYCN*-amplification with better patient outcome. Furthermore, overexpression of *EPAS1*/HIF2α in *MYCN*-amplified neuroblastoma cells leads to a rapid depletion of MYCN protein, followed by a significant reduction in proliferation rate and induction of a transcriptional response reflecting gene expression in the noradrenergic chromaffin lineage of the developing adrenal medulla. In a xenograft model of neuroblastoma, induction of HIF2α in tumors that had reached an average volume of 500 mm^3^, efficiently impeded tumor growth. Analysis of the excised tumors revealed an absence of HIF2α immunoreactivity in certain regions, which coincided with high immunoreactivity for the cell cycle marker Ki67 and low TH staining, emphasizing the inverse relationship between high levels of HIF2α and neuroblastoma tumor growth as well as indicating that the suppressive effect of *iHIF2a* on tumor growth could be underestimated.

The reduction in MYCN protein levels occurs rapidly, already visible after 4 h of doxycycline treatment. This suggests that HIF2α may facilitate the targeting of MYCN for proteasomal degradation, which is supported by the relative stabilization of MYCN protein levels upon treatment with the proteasome inhibitor MG132. Interplay between factors involved in proteasome targeted degradation of MYCN might potentially contribute to the brief recovery of MYCN protein levels at 8 h (Fig. 4*C*). Given the central role for MYCN and its target genes in high-risk neuroblastoma, the abrupt and potent depletion of MYCN upon HIF2α induction strongly argues against an oncogenic role for HIF2α in this particular disease.

The results in this study are consistent with an earlier study wherein dual treatment of neuroblastoma cells with the demethylating agent 5-Aza-2’-deoxycytidine and retinoic acid inhibited xenografted tumor growth while inducing *EPAS1* expression along with several HIF2α target genes (6). Notably, under those conditions there was a distinct reduction in *MYCN* gene expression, together with the partial restoration of MYCN levels upon PT2385 treatment (Fig. 2*M*), this implies that under certain conditions high levels of HIF2α impinges on the expression of *MYCN.* Despite the rapid reduction of MYCN protein levels, our sequence analysis revealed no reduction in *MYCN* expression upon *iHIF2α* induction after 12 or 96 h. This could either be explained by HIF2α directly affecting MYCN stability or by secondary effects, involving MYCN target genes. Alternative explanations and combinations of effects are also possible, hence the mechanisms through which HIF2α regulates MYCN levels warrant further studies.

The reduction in MYCN levels is followed by a rapid upregulation of noradrenergic markers and cell cycle exit, i.e. events reflecting the acquisition of a more mature cellular state. Whether it is the downregulation of MYCN per se that triggers this differentiation or whether lower levels of MYCN creates a transcriptional environment more amenable to signals inducing differentiation remain to be fully determined. However, the oncogenic impact of MYCN in neuroblastoma is hard to underestimate and the profound reduction in MYCN levels is likely a key factor in the cellular response to the induction of *iHIF2α*.

The high levels of *EPAS1* expression in the chromaffin lineage during adrenal medulla development potentially reflect a critical role of HIF2α in the development of catecholamine-secreting cells in the sympathoadrenal lineage, more specifically for the noradrenergic N-type rather than adrenergic E-type cells. Interestingly, HIF2α has been shown to be necessary for differentiation of neural crest derived catecholamine producing cells in mice (31) and for differentiation in the vertebrate CNS (42) as well as being able to induce a *Pnmt^−^*/*Epas1^+^*/*Rgs5^+^* noradrenergic phenotype in the mouse adrenal medulla (41). In a recent study from Adameyko and colleagues, the development of the chromaffin cell lineage in mice is described in detail (22). Two end states of differentiation are delineated with the main difference between the two subtypes being the expression of *Pnmt* i.e. E-type cells (*Th*^+^/*Dbh*^+^/*Pnmt^+^*) producing adrenaline and N-type cells (*Th*^+^/*Dbh*^+^/*Pnmt^−^*) producing noradrenaline. In addition, they identify a third terminally differentiated population expressing high levels of *Epas1*, which they hypothesize represents a mature group of oxygen sensing chromaffin cells. To our knowledge, the human chromaffin cell lineage has not been analyzed with the same resolution and there are discrepancies in marker expression between mouse and human chromaffin populations, so if a similar tripartite organization exists in humans is not known. There is some overlap between genes upregulated by *iHIF2α* induction and different genes defining the three of the chromaffin cell groups identified in the study from Adameyko and colleagues, but there is no definite pattern that perfectly fits any of the populations. We do not fully understand the role of HIF2α for chromaffin differentiation. However, the clear correlation between N-type cells and high levels of *EPAS1* as well as the upregulation of genes highly expressed in N-type cells but not the *PNMT* gene upon *iHIF2α* induction in neuroblastoma cells suggest a role for HIF2α in the generation of noradrenergic chromaffin cells but not adrenergic chromaffin cells.

There is a possibility that prolonged activation of a hypoxic transcriptional response via HIF2α, could impose a metabolic burden or cause stress in neuroblastoma cells under normoxic conditions but instead be adaptive under hypoxia. While our xenograft model supports an antiproliferative role for HIF2α even *in vivo,* future studies in additional xenograft models will be important to dissect context-dependent HIF2α effects. Interestingly, in a previous study the different roles of HIF1α and HIF2α in neuroblastoma with regard to MYCN status and hypoxia was addressed (8). Notably, the main conclusion from that study was that it was HIF1α which was predominantly expressed in *MYCN*-amplified neuroblastoma cells and primary tumors. In addition, it was HIF1α rather than HIF2α that in conjunction with MYCN was required for the Warburg effect. Thus, it appears that neuroblastoma tumors are able to cope with hypoxic stress largely due to the combinatorial effects of HIF1α and MYCN, rather than to HIF2α expression.

In a recent study on the role of the tumor suppressor gene *VHL*, Kaelin and colleagues show that upon inactivation of *VHL,* the HIF2α protein levels rise (43). This results in substantially reduced cell fitness in neuroendocrine cancer cells and induction of *NDRG1*, a member of MYCN downregulated gene family. Importantly this also substantially attenuates tumor formation in xenografts of Kelly neuroblastoma cells. Even though this study focuses on the regulation of HIF2α by VHL in another context, it shows that high levels of HIF2α is not compatible with neuroendocrine tumor cell fitness.

It is important to note that oncogenic activity of HIF2α has only been clearly demonstrated in a limited number of tumor types, most notably in clear cell renal cell carcinoma (ccRCC) and paragangliomas (PPGL). These tumors are often associated with VHL mutations, which are absent in neuroblastoma and only observed in a restricted set of tumors associated with VHL syndrome (44). Neuroblastoma is not part of the clinical spectrum of VHL-associated neoplasms. This raises the possibility that tissues predisposed to tumorigenesis upon VHL loss, such as the kidney and carotid body or chromaffin tissue, may be intrinsically adapted to hypoxia and oxygen-sensing mechanisms, making them uniquely permissive to the oncogenic effects of HIF2α stabilization.

Furthermore, although VHL mutations are found in the vast majority of ccRCCs (~90%) and in the hypoxic subtype of PPGL, HIF2α mutations (*EPAS1*) are rare overall, not seen in ccRCC, and typically only occur as somatic mutations (not germline) in a subset of PPGLs (44). This suggests that HIF2α-driven tumorigenesis may be tolerated only in certain cellular contexts, possibly due to specific epigenetic, metabolic, or developmental properties of those tissues. This remarkable cancer-type specificity of HIF2α oncogenic activity reinforces the view that its role is highly context-dependent, and that in neuroblastoma, lacking VHL mutations, HIF2α may serve a different, potentially tumor-suppressive function.

Our study has not addressed all potential aspects of neuroblastoma and we cannot exclude the possibility that HIF2α can be involved in processes such as metastasis, relapse, drug-resistance, or in hypoxia-associated events affecting tumor pathophysiology. However, our computational analysis of several neuroblastoma and adrenal medulla datasets combined with functional experiments in vitro and in vivo preclinical models of neuroblastoma suggest that HIF2α is not an oncogene in neuroblastoma but potentially harbors tumor suppressive characteristics, which are probably connected to the reduction of MYCN protein levels. Despite a validated role as an oncogene in renal cell carcinoma (4), HIF2α has been shown to exhibit tumor suppressive properties in several other types of cancers (45–52). Given these differences it is feasible that the role of HIF2α is highly context dependent. Indeed, in models of non–small lung cancer it has been reported that HIF2α can act both as a facilitator and inhibitor of tumor growth (49, 53). Hence, it cannot be completely ruled out that even within the same type of tumor, a certain “goldilocks” level of HIF2α might have oncogenic impact for a distinct cellular state but that levels of HIF2α outside of such a zone are tumor suppressive. A similar “goldilocks” model has previously been suggested for paragangliomas and pancreatic neuroendocrine tumors (43, 44, 54). However, such a role for HIF2α in neuroblastoma remains to be shown, potentially in experimental models wherein HIF2α levels can be further controlled both spatially and temporally. The substantial induction of genes typical of noradrenergic differentiation does not translate directly to a tumor suppressive role but in combination with reduced proliferation, reduced MYCN levels, impaired tumor growth and the pattern in several hundreds of tumors as well as several thousand single tumor cells from patients, it challenges the predominant view that HIF2α is an oncogene. Yet, further studies are required to resolve the full role of HIF2α in neuroblastoma.

## Materials and Methods

Human neuroblastoma (NB) cell lines LAN-1, SK-N-BE(2), Kelly, and SH-EP2, together with the renal carcinoma line 786-O, were cultured at 37 °C in a humidified atmosphere of 95% air and 5% CO_2_. Cells were maintained in RPMI-1640 medium (Gibco, 31870) supplemented with 10% fetal bovine serum (HyClone, SV30180), 2 mM L-glutamine (Gibco, 25030), 100 U mL^−1^ penicillin, and 100 µg mL^−1^ streptomycin (Gibco, 15140). Cultures were passaged every 2 to 3 d to preserve exponential growth.

Human umbilical vein endothelial cells (HUVEC; ATCC CRL-4053) were propagated under identical incubation conditions in Endothelial Cell Growth Medium MV2 (PromoCell, C-22022) supplemented with 100 U mL^−1^ penicillin, and 100 µg mL^−1^ streptomycin.

HUVECs were generously provided by the Samir El Andaloussi laboratory, whereas Kelly and SH-EP2 cells were gifts from the Marie Arsenian-Henriksson laboratory.

### Western Blot Analysis.

Upon completion of Dox treatment, LAN-1 and SK-N-BE(2) cells were collected and lysed with ice-cold EBC lysis solution (50 mM Tris, pH 8.0, 120 mM NaCl, 0.5% Nonidet P-40). Protein concentration was measured through Bradford assay (Biorad). Equal amounts of proteins were loaded on 10% SDS PAGE, followed by transfer on nitrocellulose membrane at 90 V for 1.5 h. Following transfer, the membrane was blocked with 5% Skim milk in 1x Phosphate Buffer Saline+0.5% Tween20 (PBST) for 30 min. After 30 min, membranes were blotted with primary antibodies dissolved in PBST overnight in cold room. Next day, the membranes were incubated with anti-mouse or anti-rabbit IgG conjugated secondary antibody solution for 1.5-2 h and then developed with ECL immobilon western chemiluminescent HRP substrate (Millipore, WBKLS0500). Images were captured by Amersham and LAS4000 imaging systems. Membranes were then reblotted with either GAPDH (1:3000) or β-ACTIN (1:3000) next day for internal normalization.

### Immunostainings.

Following completion of treatment, equal number of cells (grown on coverslips) were fixed with 4% PFA in room temperature for 20 min. Gradual fixation with 2% PFA for 5 min and then 4% PFA for 15 min was done for LAN-1 and SH-SY5Y cells which were kept in long-term culture. Following fixation, cells were blocked with 5% BSA, 0.2% Tween 20, and 0.02% sodium azide in 1X PBS solution for one hour, followed by overnight antibody incubation in cold condition. Next day, cells were incubated with a combination of DAPI and appropriate Alexa fluorophore conjugated secondary (anti-mouse A2102, anti-Rabbit A31572, Thermofisher scientific) antibody solutions for 90 min. Afterward cover slips were mounted on slides. Images were acquired in Leica SP8 confocal microscopy at 40x zoom, 1024x1024 dimension.

### Vectors and Stable Cell Lines Construction.

The HA-HIF2α-P405A/P531A-pBabe-puro plasmid was generously provided by the Susanne Schlisio laboratory. The PiggyBac transposase vector pCAG T7 K hyPBase was a gift from the Kenneth Chien laboratory. Primers for cloning were designed, and the HA-HIF2α-P405A/P531A construct was inserted into a custom-made inducible PiggyBac Transposon vector, pB-TRE-Luc2. All cloned sequences were verified by Sanger sequencing, performed by Integrated DNA Technologies (IDT).

To generate stable cell lines, the transposon vectors pB-TRE-HA-HIF2α-P405A/P531A-LUC2 or pB-TRE-LUC2, along with the transposase vector pCAG-T7 K hyPBase, were cotransfected into cells using Lipofectamine 2000 (Invitrogen) at a 4:1 vector-to-transposase ratio. Forty-eight hours posttransfection, cells were selected in 200 μg/mL Hygromycin B until nontransfected control cells were completely eliminated. This approach successfully established an inducible, stable HIF2α expression system, functional even under normoxic conditions.

Cells were seeded either directly into culture dishes or on top of coverslips placed in dishes at approximately 50% confluence. The following day, doxycycline (Clontech Laboratories) was added to the culture medium at a final concentration of 50 ng/mL for LAN-1 cells, and 250 ng/mL for SK-N-BE(2) and SH-EP2 cells, along with either 10 μM of PT2385 (Sigma-Aldrich), for the designated treatment duration. The medium containing doxycycline and PT2385 was replenished every 2 d. At the conclusion of the experiment, cells were either fixed with 4% paraformaldehyde (PFA, Sigma-Aldrich) or harvested for subsequent experimental analyses.

### RNA Sequencing and Data Analysis.

Total RNA was extracted from cultured cells or xenografted tumor tissues using the RNeasy Kit (Qiagen) according to the manufacturer’s instructions. RNA quality and quantity were assessed using a Bioanalyzer (Agilent Technologies) to ensure high-quality RNA input. RNA sequencing (RNA-seq) libraries were prepared using the TruSeq RNA Library Preparation Kit v2 (Illumina) following the standard protocol. Paired-end RNA sequencing was performed by Novogene, and high-throughput sequencing data were processed for quality control, alignment, and differential expression analysis using a combination of established bioinformatics tools, such as FastQC, STAR, and DESeq2. Gene expression profiles were further analyzed for pathway enrichment and functional annotation.

### Statistical Analysis.

For the analysis in Fig. 1 *J*–*M* expression data normalized and standardized was kindly provided by Bedoya-Reina et al. 2021 and correspond to that obtained with the PAGODA pipeline. Plots were done using Scanpy. For significance calculations in Fig. 1*M*, Welch’s *t* test was used. Cell cycle data in Fig. 2*N* and *SI Appendix*, Fig. S5*Q* were analyzed using ANOVA with Tukey’s multiple comparisons test. Xenograft growth in Fig. 6*A* was analyzed with two-way ANOVA, with Sídák’s multiple comparisons test and tumor weight in Fig. 6*B* was analyzed using the unpaired t test with Welch’s correction.

### Lentiviral Transduction and Puromycin Selection.

Cells were transduced with virus particles purchased from VectorBuilder carrying the following constructs: pLV[Exp]-EGFP/Puro-EF1A > hASCL1, pLV[Exp]-EGFP/Puro-EF1A > hPHOX2B, pLV[Exp]-EGFP/Puro-EF1A > hHIF1A, and pLV[Exp]-EGFP/Puro-EF1A > mCherry or from Vector Biolabs carrying the following constructs: Ad-CMV-GFP, Ad-CMV-GFP-hEPAS1. Cells were seeded at ~50 to 60% confluency and infected with the respective viral constructs at a multiplicity of infection (MOI) of 5 for LAN-1, 10 for SH-SY5Y, and 2 for Kelly cells. Transductions were carried out in the presence of 8 μg/mL polybrene (VectorBuilder) to enhance infection efficiency. After 24 h, the medium was replaced with fresh complete growth medium. Puromycin (Gibco) selection was initiated 48 h posttransduction using concentrations of 0.5 μg/mL. Selection was continued for 4–6 d until uninfected control cells were fully eliminated. Surviving puromycin-resistant cells were expanded and used for downstream analyses.

### Gene Module Scores Analysis.

Gene module scores were computed using the *AddModuleScore* function from the Seurat R package, version V5.0.0 (55). The reference dataset for the analysis, encompassing embryonic adrenal medulla data, was obtained from Jansky et al. (available at: https://adrenal.kitz-heidelberg.de/developmental_programs_NB_viz/). To identify genes for module score calculation, differentially expressed genes (DEGs) were filtered based on the absolute log2FoldChange values derived from DEG analysis. Two gene sets were created by applying less stringent (|log2FoldChange| > 2) and high-stringency (|log2FoldChange| > 5) thresholds, respectively. These filtered gene lists were subsequently used as input gene modules for the *AddModuleScore* function in Seurat, generating module scores for each individual cell. The resulting scores were added to the reference dataset as additional metadata.

### Mouse Xenografts.

A suspension of 2 × 10^6^ neuroblastoma cells in 200 μL of a 1:1 mixture of phosphate-buffered saline (PBS) and Matrigel (BD Biosciences, 354248) was injected subcutaneously into the right flank of adult female Crl (Ncr)-Foxn1^nu^ (nude) mice. Tumor growth was monitored by measuring the external dimensions using a digital caliper every other day. The greatest longitudinal diameter (length) and the greatest transverse diameter (width) were recorded, and tumor volume was calculated using the modified ellipsoid formula: Tumor volume= [(length × width^2^)/2].

Mice were provided with a doxycycline-supplemented diet (0.625 g/kg Doxycycline Hyclate) once the tumors reached an approximate volume of 500 mm^3^. The doxycycline diet was replenished every 2 d. Throughout the experiment, overall health status was carefully monitored, and body weight was measured weekly. At the end of the study, mice were killed in accordance with approved protocols, and tumors were excised. Tumor weights were measured, and the samples were snap-frozen for subsequent analyses.

### Immunofluorescence Staining on Xenograft Cryosections.

Xenograft tumors were excised at the endpoint of the experiment and immediately snap-frozen in optimal cutting temperature (OCT) compound (Sakura Finetek) using liquid nitrogen. Frozen tumors were sectioned into 8 to 10 μm thick slices using a cryostat (Leica), and the sections were mounted onto Superfrost Plus glass slides (ThermoFisher Scientific). Slides were air-dried at room temperature for 30 min before being fixed in cold 4% paraformaldehyde (PFA) for 15 min at 4 °C. Following fixation, the sections were washed three times in phosphate-buffered saline (PBS) and permeabilized with 0.1% Triton X-100 in PBS for 10 min. Nonspecific binding was blocked by incubating the sections in PBS containing 5% normal goat serum (NGS) or normal donkey serum (NDS) for 1 h at room temperature. Primary antibodies were diluted in PBS with 1% NGS or NDS and applied to the sections, followed by incubation overnight at 4 °C in a humidified chamber. After incubation with primary antibodies, the sections were washed three times with PBS and incubated with fluorophore-conjugated secondary antibodies (Invitrogen) together with DAPI (4’,6-diamidino-2-phenylindole, Sigma-Aldrich) for 1 h at room temperature in the dark. After wash with PBS, slides were mounted with ProLong Gold Antifade Mountant (Thermo Fisher Scientific) and coverslipped. Immunofluorescence images were acquired using a confocal laser scanning microscope (Zeiss, LSM700).

### Ethical Considerations.

All animal experiments were performed according to Swedish guidelines and regulations, the ethical permit 6420 to 2018 was granted by ‘Stockholms Norra djurförsöksetiska nämnd, Sweden.

Primary neuroblastoma (NB) tumor samples were obtained from the Swedish Neuroblastoma Registry, with ethical clearance (DN03-736) provided by the Regional Ethical Review Board at Karolinska Institutet, Stockholm, Sweden. Prior to sample collection, written informed consent was obtained from the families of all subjects, in compliance with ethical standards and data protection regulations.

## Supplementary Material

Appendix 01 (PDF)

Dataset S01 (XLSX)

Dataset S02 (XLSX)

Dataset S03 (XLSX)

Dataset S04 (XLSX)

Dataset S05 (XLSX)

Dataset S06 (XLSX)

Dataset S07 (XLSX)

Dataset S08 (XLSX)

Dataset S09 (XLSX)

## Data Availability

RNA sequencing data are available at the NCBI BioProject database under the accession numbers PRJNA1332815 (56) and PRJNA1333560 (57).
