# Supplementary material for: HIF2α negatively regulates MYCN protein levels and promotes a low-risk noradrenergic phenotype in neuroblastoma
Source: Proc Natl Acad Sci U S A. 2025 Oct 21;122(43):e2516922122. doi: 10.1073/pnas.2516922122 (PMC12582314; doi:10.1073/pnas.2516922122)
Supplement: Supplementary file 1 — Appendix 01 (PDF) [file pnas.2516922122.sapp.pdf]

S1

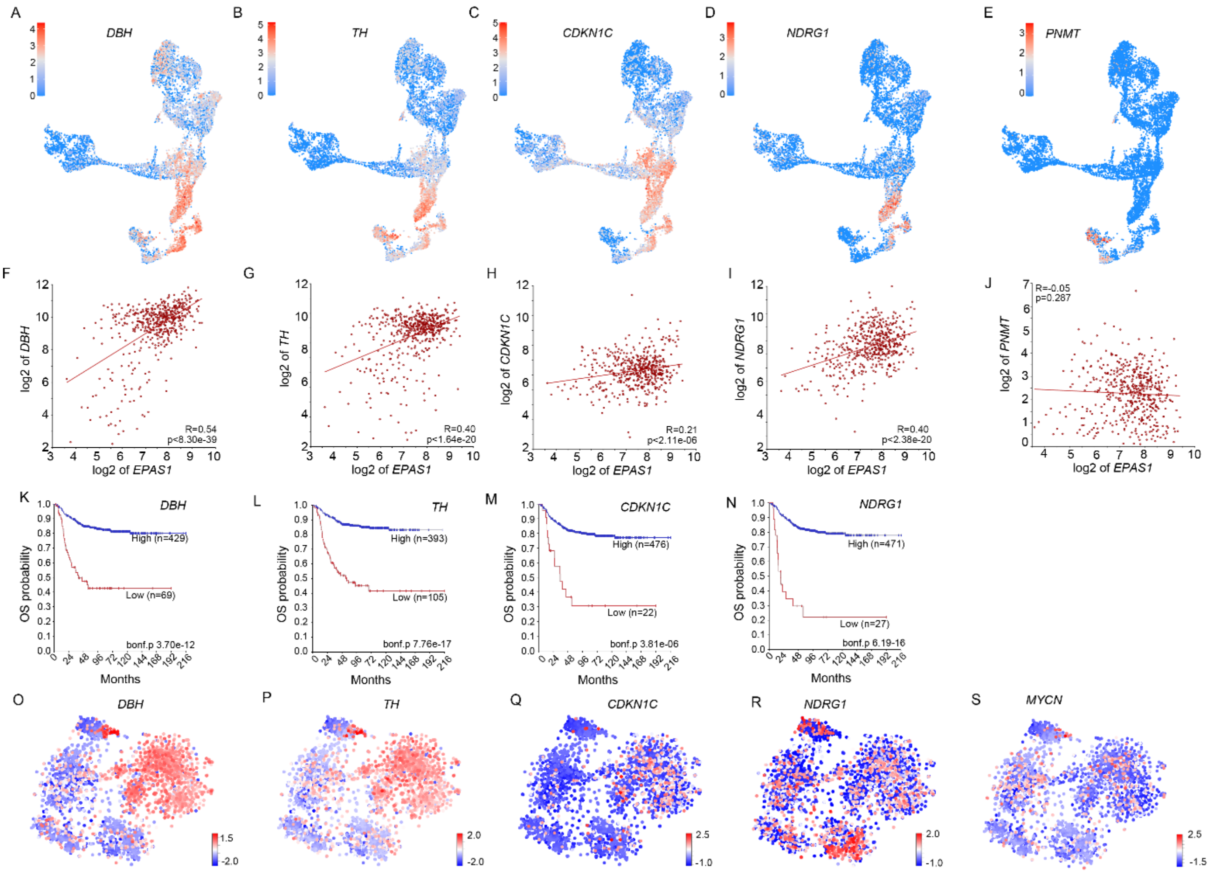

Supplementary Figure S1. *EPAS1* expression is associated with the chromaffin cell lineage, *cyclin dependent kinase inhibitor 1C,* and the *NDRG1* gene downregulated by MYCN.

(A-D) *DBH* (A), *TH* (B), *CDKN1C* (C) and *NDRG1* (D) expression are enriched in connecting progenitor and chromaffin cells in the developing human adrenal medulla, dataset from Jansky et al.

(E) *PNMT* expression is enriched in late chromaffin cells.

(F) Expression of *DBH* and *EPAS1* are positively correlated in neuroblastoma tumors.

(G) Expression of *TH* and *EPAS1* are positively correlated in neuroblastoma tumors.

(H) Expression of *CDKN1C* and *EPAS1* are positively correlated in neuroblastoma tumors.

(I) Expression of *NDRG1* and *EPAS1* are positively correlated in neuroblastoma tumors.

(J) Expression of *PNMT* and *EPAS1* are not correlated in neuroblastoma tumors.

(K) *DBH* expression is correlated to increased overall survival.

(L) *TH* expression is correlated to increased overall survival.

(M) *CDKN1C* expression is correlated to increased overall survival.

(N) *NDRG1* expression is correlated to increased overall survival.

(O) Mapping of *DBH* expression in tSNE of neuroblastoma single cell nuclei show enrichment in noradrenergic low-risk tumor cells, dataset from Bedoya-Reina et al.

(P) *TH* expression is enriched for in noradrenergic low-risk tumor cells.

(Q) *CDKN1C* expression is enriched for in noradrenergic low-risk tumor cells.

(R) *NDRG1* expression is enriched for in endothelial cells.

(S) *MYCN* expression.

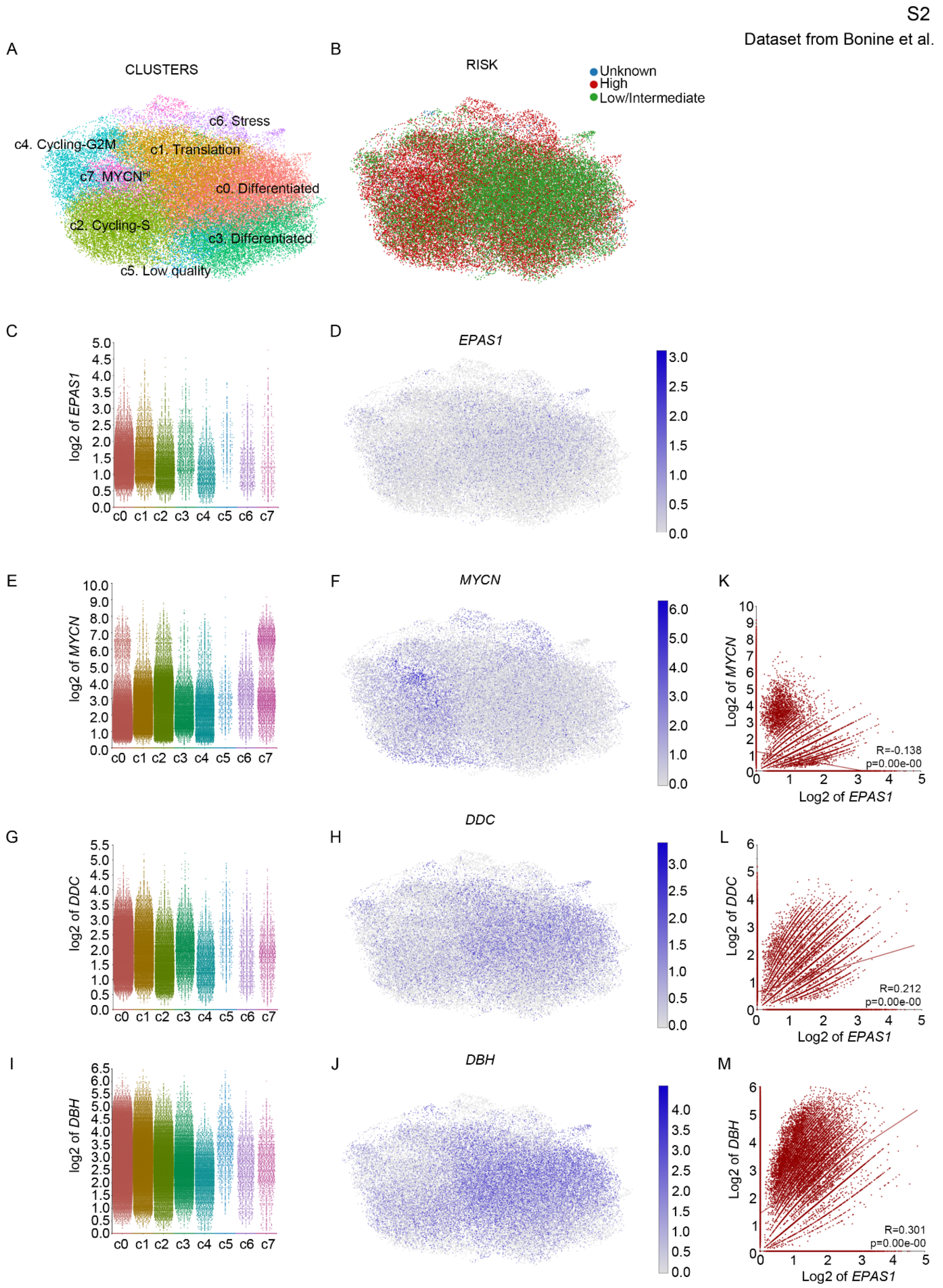

Supplementary Figure S2. *EPAS1*, *DDC* and *DBH* expression is prominent in low-risk tumor cells with low levels of *MYCN* expression.

(A) Annotated map of malignant tumour cells (n=126872).

(B) UMAP plot showing neuroblastoma cells of different risk levels, according to Bonine et al.

(C-D) Expression of *EPAS1* in the annotated clusters (C) and in the total cell population (D).

(E-F) Expression of *MYCN* in the annotated clusters (E) and in the total cell population (F).

(G-H) Expression of *DDC* in the annotated clusters (G) and in the total cell population (H).

(I-J) Expression of *DBH* in the annotated clusters (I) and in the total cell population (J).

(K-M) Correlation between *EPAS1* expression and *MYCN* (K), *DDC* (L) and *DBH* (M) expression.

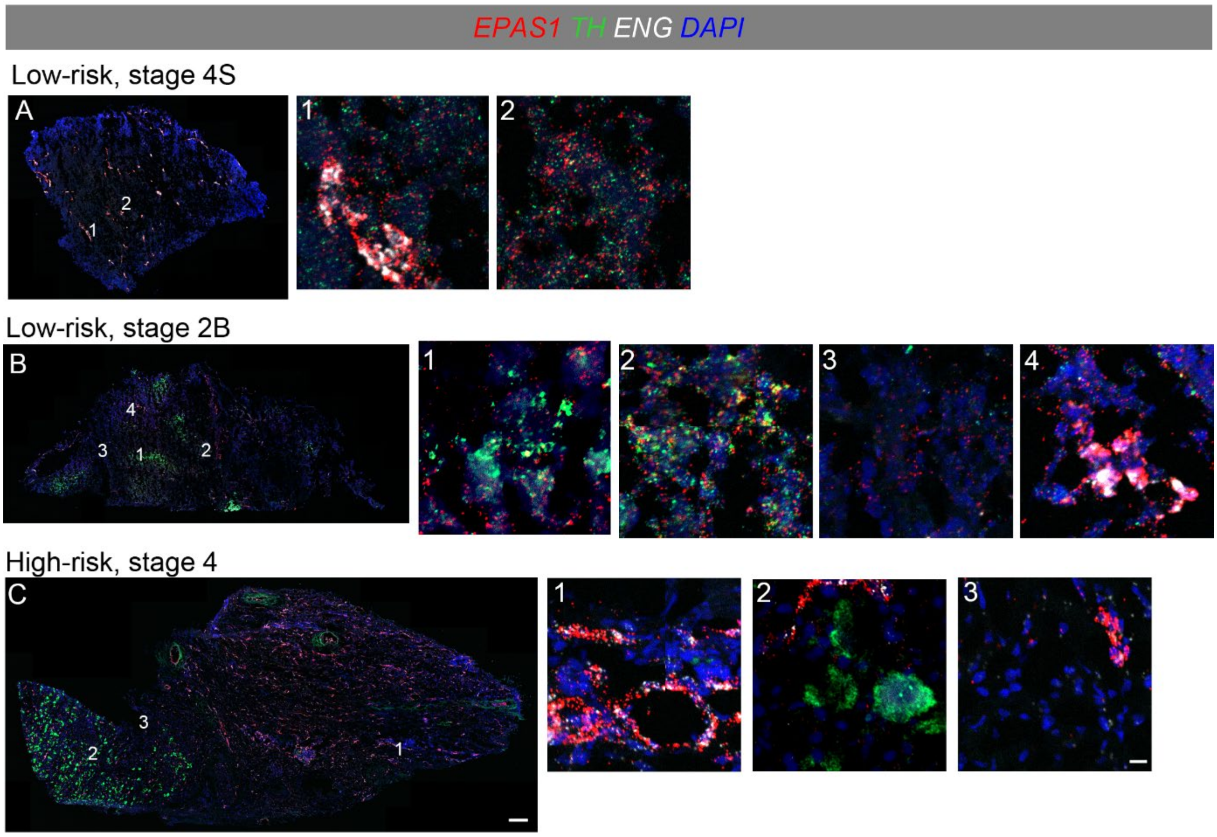

Supplementary Figure S3. Tiled-scanned 20x images of RNAscope *in situ* hybridization showing *EPAS1, TH* and *ENG* expression in neuroblastoma tumours of different stages.

RNAscope with probes for *EPAS1* (red), *TH* (green), *ENG* (Blue) and DAPI in white to visualize nuclei.

(A) Low-risk, stage 4S tumour, 1 and 2 represent zoomed in regions as indicated.

(B) Low-risk, stage 2B tumour, 1, 2, 3 and 4 represent zoomed in regions as indicated.

(C) High-risk, stage 4 tumour, 1, 2 and 3 represent zoomed in regions as indicated.

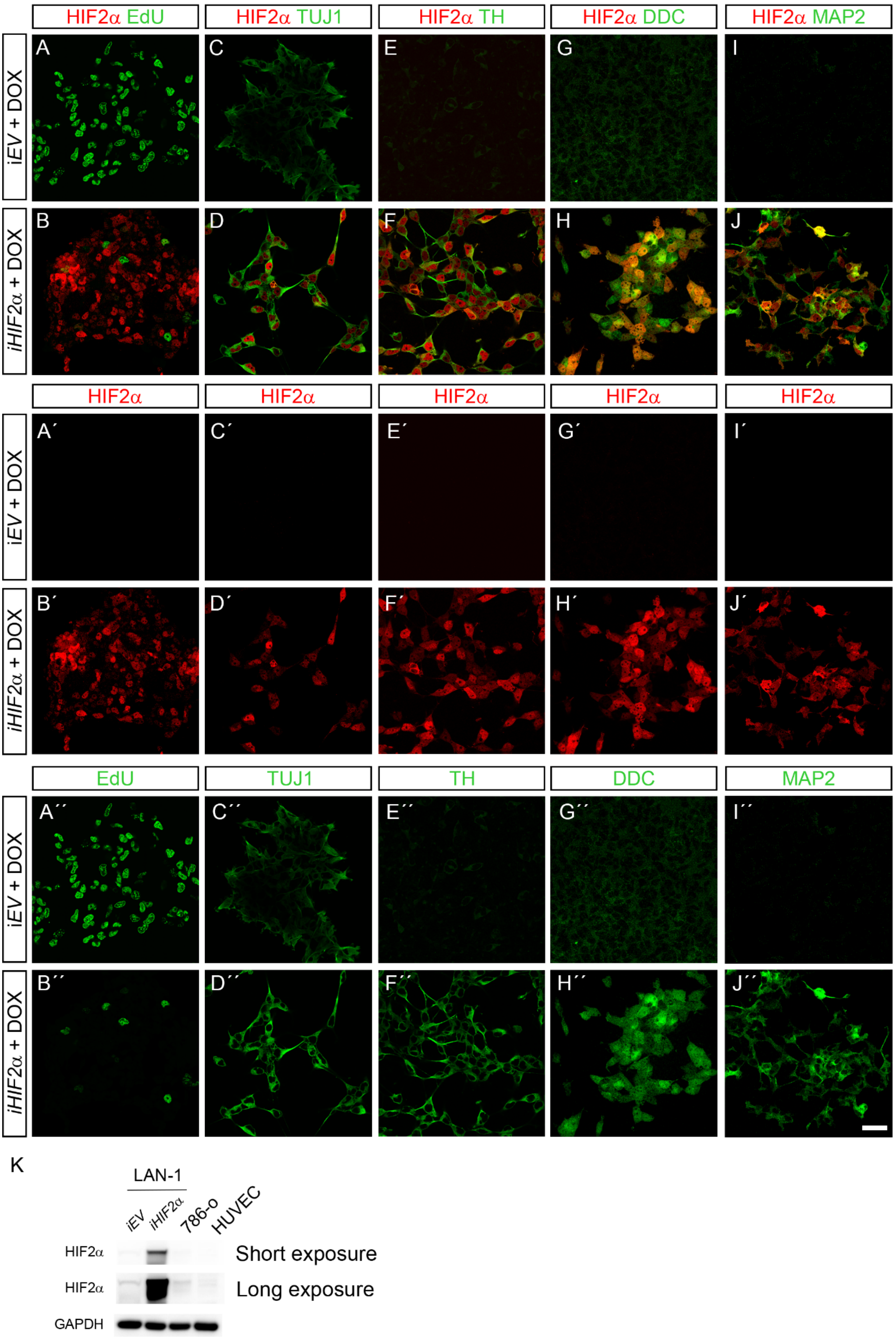

HIF2α EdU
HIF2α TUJ1
HIF2α TH
HIF2α DDC
HIF2α MAP2
iEV + DOX
iHIF2α + DOX
A
B
C
D
E
F
G
H
I
J
HIF2α
A´
B´
C´
D´
E´
F´
G´
H´
I´
J´
EdU
TUJ1
TH
DDC
MAP2
A´´
B´´
C´´
D´´
E´´
F´´
G´´
H´´
I´´
J´´
K
LAN-1
iEV
iHIF2α
786-o
HUVEC
HIF2α
Short exposure
HIF2α
Long exposure
GAPDH

Supplementary Figure S4. Overexpression of HIF2α in *MYCN*-amplified LAN-1 neuroblastoma cells leads to reduced proliferation and upregulation of chromaffin associated factors. Related to Fig. 2C-L).

(A-J) Immunostaining with HIF2α and the indicated antibody in *iEV* or *iHIF2a* LAN-1 cells.

(A´-J´) Identical staining as in A-J but only showing the red channel.

(A´´-J´´) Identical staining as in A-J but only showing the green channel.

(K) Western blot for comparison of HIF2α protein levels in *iHIF2α* induced LAN-1 cells with 786-o and HUVEC cells. GAPDH is shown as loading control.

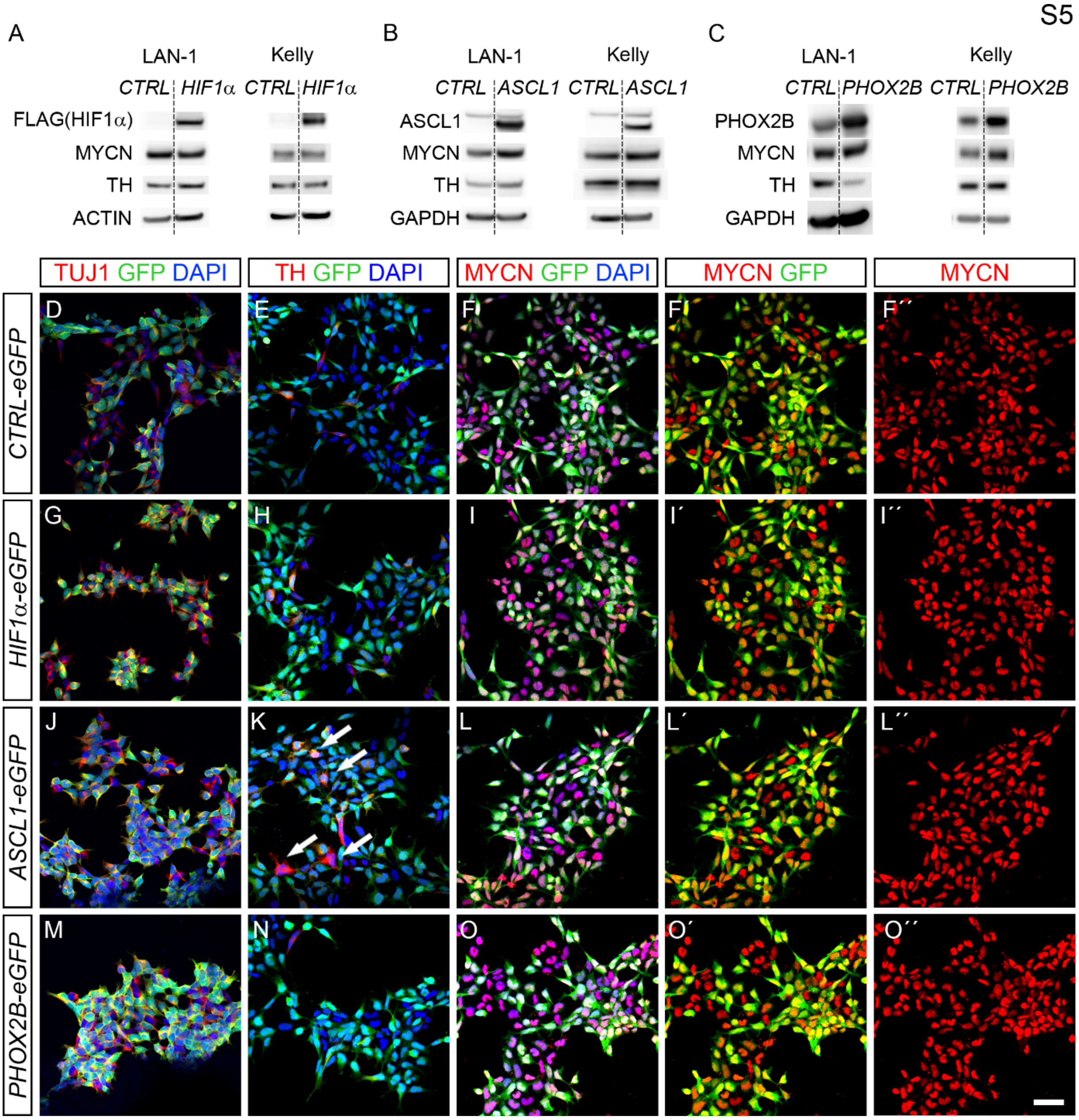

Supplementary Figure S5. Overexpression of *HIF1α, ACSL1* or *PHOX2B* in *MYCN*-amplified neuroblastoma cells.

(A-C) Western blot with the indicated antibodies after overexpression of *HIF1α* (A), *ASCL1* (B) and *PHOX2B* (C) in LAN-1 and Kelly neuroblastoma cells.

(D-F´´) Immunostaining with the indicated antibodies in LAN-1 cells overexpressing the *CTRL-eGFP* construct, F´and F´´ identical microscopic field as F.

(G-I´´) Imunostaining with the indicated antibodies in LAN-1 cells overexpressing the *HIF1α-eGFP* construct, I´and I´´ identical microscopic field as I.

(J-L´´) Immunostaining with the indicated antibodies in LAN-1 cells overexpressing the *ASCL1-eGFP* construct, L´and L´´ identical microscopic field as L. Arrows in K indicating GFP^{+}-cells wherein TH protein levels are increased.

(M-O´´) Immunostaining with the indicated antibodies in LAN-1 cells overexpressing the *CTRL-eGFP* construct, O´and O´´ identical microscopic field as O.

Scale bar in O´´=50μm.

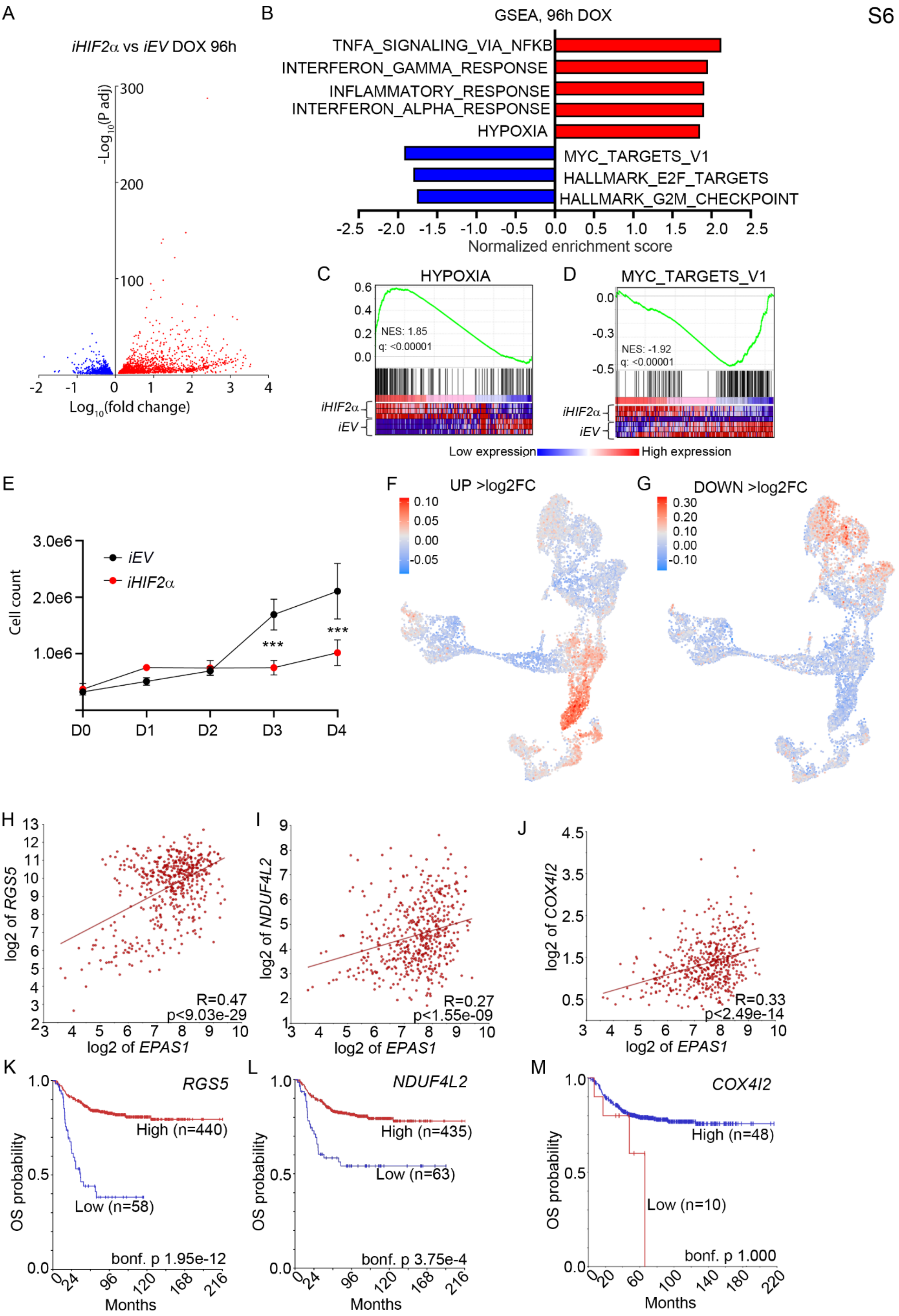
S6
A
iHIF2α vs iEV DOX 96h
-Log10(P adj)
Log10(fold change)
B
GSEA, 96h DOX
TNFA_SIGNALING_VIA_NFKB
INTERFERON_GAMMA_RESPONSE
INFLAMMATORY_RESPONSE
INTERFERON_ALPHA_RESPONSE
HYPOXIA
MYC_TARGETS_V1
HALLMARK_E2F_TARGETS
HALLMARK_G2M_CHECKPOINT
Normalized enrichment score
C
HYPOXIA
NES: 1.85
q: <0.00001
D
MYC_TARGETS_V1
NES: -1.92
q: <0.00001
iHIF2α
iEV
Low expression
High expression
E
iEV
iHIF2α
Cell count
F
UP >log2FC
G
DOWN >log2FC
H
log2 of RGS5
log2 of EPAS1
R=0.47
p<9.03e-29
I
log2 of NDUF4L2
log2 of EPAS1
R=0.27
p<1.55e-09
J
log2 of COX4I2
log2 of EPAS1
R=0.33
p<2.49e-14
K
RGS5
High (n=440)
Low (n=58)
bonf. p 1.95e-12
OS probability
Months
L
NDUF4L2
High (n=435)
Low (n=63)
bonf. p 3.75e-4
M
COX4I2
High (n=48)
Low (n=10)
bonf. p 1.000

Supplementary Figure S6. After 96h overexpression of *EPAS1* causes expression of genes associated with hypoxia and the chromaffin cell lineage while there is a reduction of MYCN targets and genes associated with cell cycle progression.

(A) Volcano plot showing genes upregulated (red) and downregulated (blue) 96 hours after induction of *iHIF2*α.

(B) Gene set enrichment analysis (GSEA) of the genes from (A).

(C) GSEA of the "HYPOXIA" gene set.

(D) GSEA of the "MYC_TARGETS_V1".

(E) Growth curve over 4 days after doxycycline induction of *iEV and iHIF2*α in LAN-1 cells.

(F) Genes upregulated >Log2FC in (A) plotted on the developing adrenal medulla from Jansky et al.

(G) Genes downregulated >Log2FC in (A) plotted on the developing adrenal medulla from Jansky et al.

(H-J) Correlation between *EPAS1* expression and *RGS5* (H), *NDFU4L2* (I) and *COX4I2* (J) in the 498SEQC neuroblastoma cohort.

(K-L) *RGS5* (K) and *NDFU4L2* (L) expression are correlated with increased overall patient survival.

(M) *COX4I2* expression is not significantly corelated with patient survival.

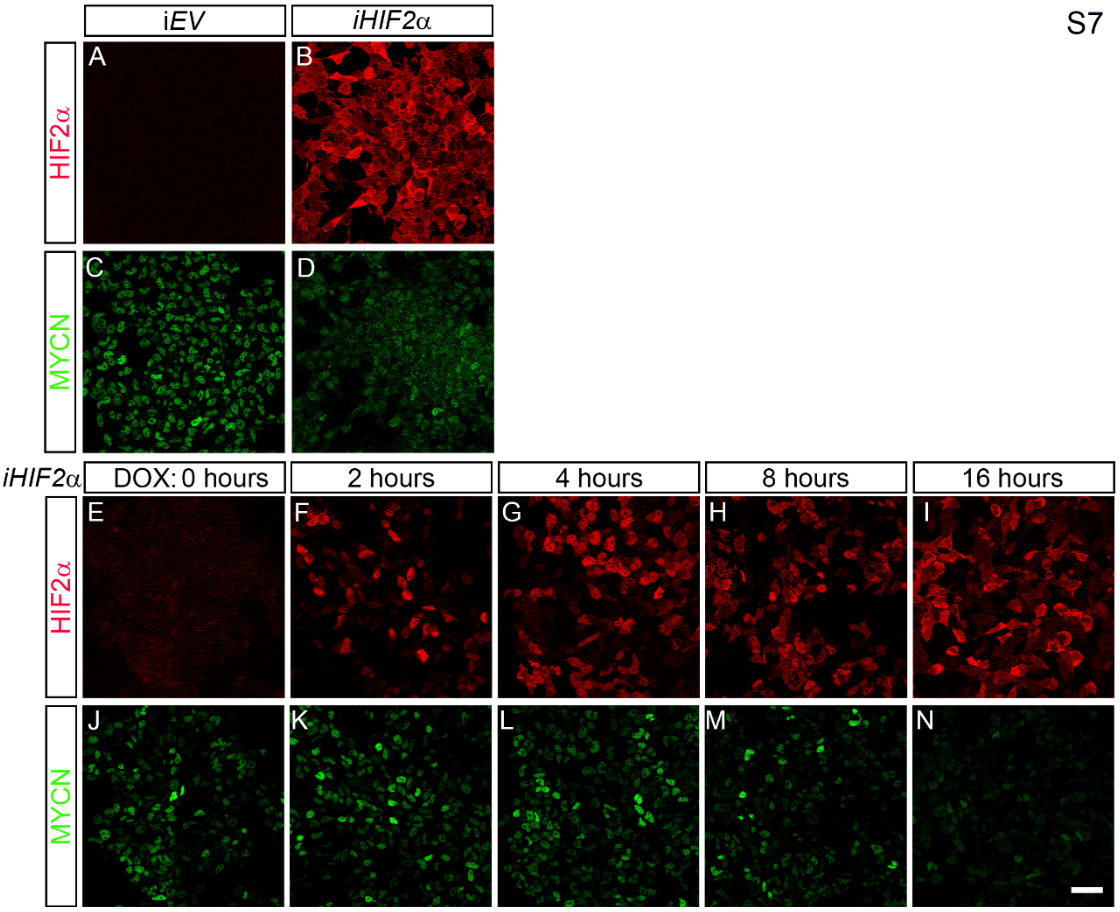

Supplementary Figure S7. Induction of *iHIF2α* depletes MYCN protein levels. Similar panels as in Fig. 4 A-B and D-H but in single channels.

(A-D) In *iEV* expressing cells there is no induction of HIF2α and MYCN protein levels remain high, 24h after doxycycline treatment (A, C), whereas in *iHIF2α* cells there is an upregulation of HIF2α and an reduction in MYCN protein levels (B, D).

(E-N) Immunostaining with antibodies for HIF2α (red) (E-I) and MYCN (green) (J-N) at the indicated time points after doxycycline induction.

Scale bar in N=50μm.

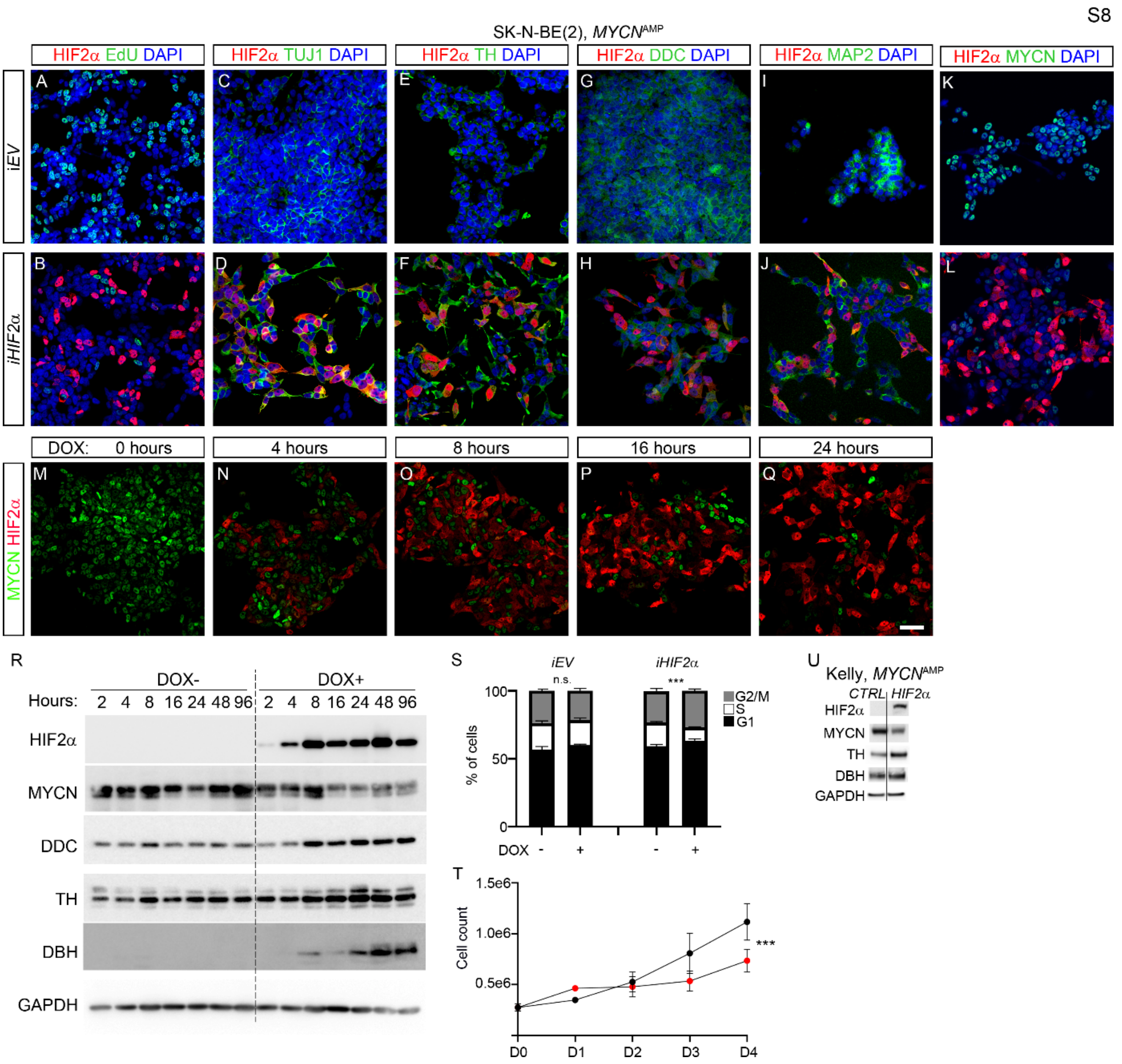

Supplementary Figure S8. Overexpression of HIF2α in *MYCN*-amplified SK-N-BE(2) neuroblastoma cells leads to reduced MYCN protein levels and upregulation of noradrenergic chromaffin cell associated factors followed by a reduction in proliferation.

(A-B) Immunostaining with HIF2α (red) combined with EdU (green) and DAPI (blue) in *iEV* control cells (A) and in *iHIF2α* (B) cells treated with doxycycline.

(C-D) Immunostaining with HIF2α (red) combined with TUJ1 (green) and DAPI (blue) in *iEV* control cells (C) and in *iHIF2α* (D) cells treated with doxycycline.

(E-F) Immunostaining with HIF2α (red) combined with TH (green) and DAPI (blue) in *iEV* control cells (E) and in *iHIF2α* (F) cells treated with doxycycline.

(G-H) Immunostaining with HIF2α (red) combined with DDC (green) and DAPI (blue) in *iEV* control cells (G) and in *iHIF2α* (H) cells treated with doxycycline.

(I-J) Immunostaining with HIF2α (red) combined with MAP2 (green) and DAPI (blue) in *iEV* control cells (I) and in *iHIF2α* (J) cells treated with doxycycline.

(I-J) Immunostaining with HIF2α (red) combined with MYCN (green) and DAPI (blue) in *iEV* control cells (I) and in *iHIF2α* (J) cells treated with doxycycline.

(M-Q) Immunostaining with antibodies for MYCN (green) and HIF2α (red) at the indicated time points after doxycycline induction.

(R) Western blot showing upregulation of HIF2α, DDC, TH, and DBH but downregulation of MYCN after doxycycline induction. GAPDH is shown as loading control.

(S) Cell cycle analysis after PI-staining shows a decrease in cells in S-phase and G2/M-phase upon doxycycline induction of *iHiF2α.*

(T) Growth curve over 4 days after doxycycline induction of *iEV and iHIF2α* in SK-N-BE(2) cells.

(U) Western blot showing protein levels of the indicated antibodies in Kelly cells either expressing *CTRL* or *HIF2α* constructs. GAPDH is shown as loading control.

Cell cycle data is represented as mean ± SD; P-value of differences in S-phases was calculated with ANOVA with Tukey's multiple comparisons test.

Scale bar in Q=50μm.

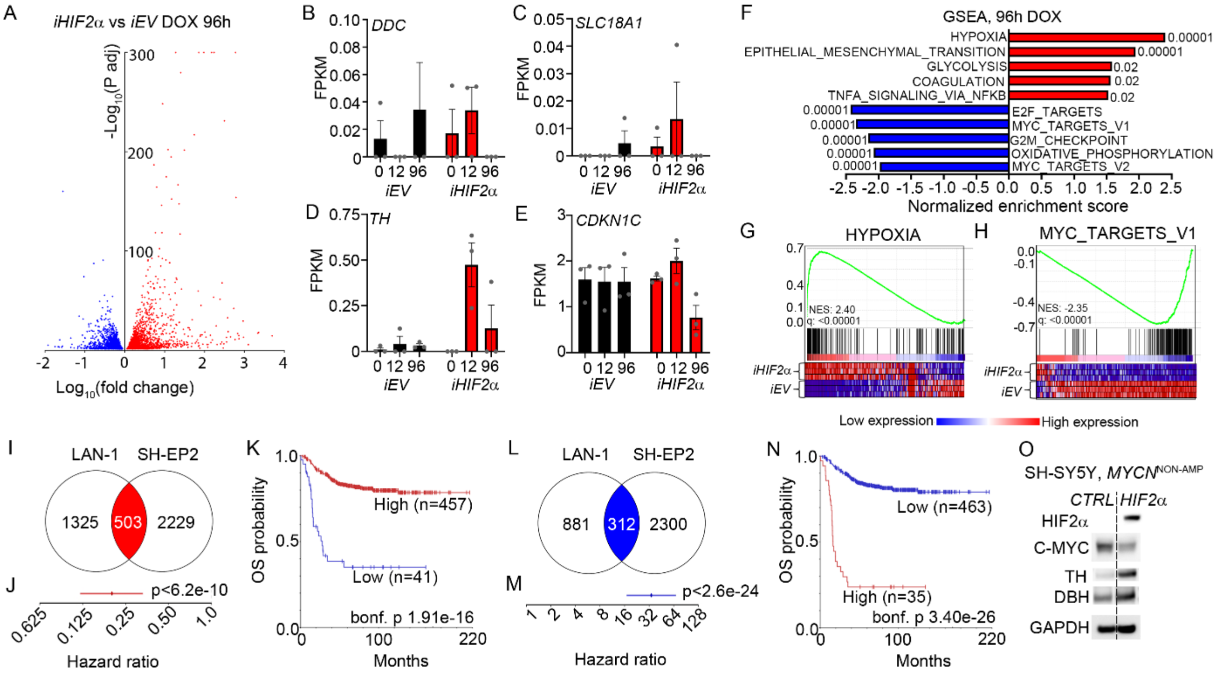

Supplementary Figure S9. Overexpression of *EPAS1* for 96h in Overexpression of HIF2α in non-*MYCN*-amplified SH-EP2 mesenchymal neuroblastoma induces expression of genes associated with hypoxia while there is a reduction of MYC targets and genes associated with cell cycle progression.

(A) Volcano plot showing genes upregulated (red) and downregulated (blue) 96 hours after induction of *iHIF2α*.

(B-E) Expression levels of the indicated genes 0, 12 and 96 hours after doxycycline induction. No significant differences were detected.

(F) Gene set enrichment analysis (GSEA) of the genes from (A), numbers indicate FDR.

(G) GSEA of the “HYPOXIA” gene set.

(H) GSEA of the “MYC_TARGETS_V1” gene set.

(I) Genes commonly upregulated in *iHIF2α* LAN-1 and SH-EP2 cells, 96 hours after doxycycline induction.

(J) Hazard ratio of commonly upregulated genes in (I), in the 498SEQC neuroblastoma cohort.

(K) Expression of commonly upregulated genes in the 498SEQ neuroblastoma cohort is correlated with increased overall survival.

(L) Genes commonly downregulated regulated in *iHIF2α* LAN-1 and SH-EP2 cells, 96 hours after doxycycline induction.

(M) Hazard ratio of commonly downregulated genes in (L), in the 498SEQC neuroblastoma cohort.

(N) Expression of commonly downregulated regulated genes in the 498SEQ neuroblastoma cohort is correlated with decreased overall survival.

(O) Western blot showing protein levels of the indicated antibodies in SH-SY5Y cells either expressing *CTRL* or *HIF2α* constructs. GAPDH is shown as loading control.

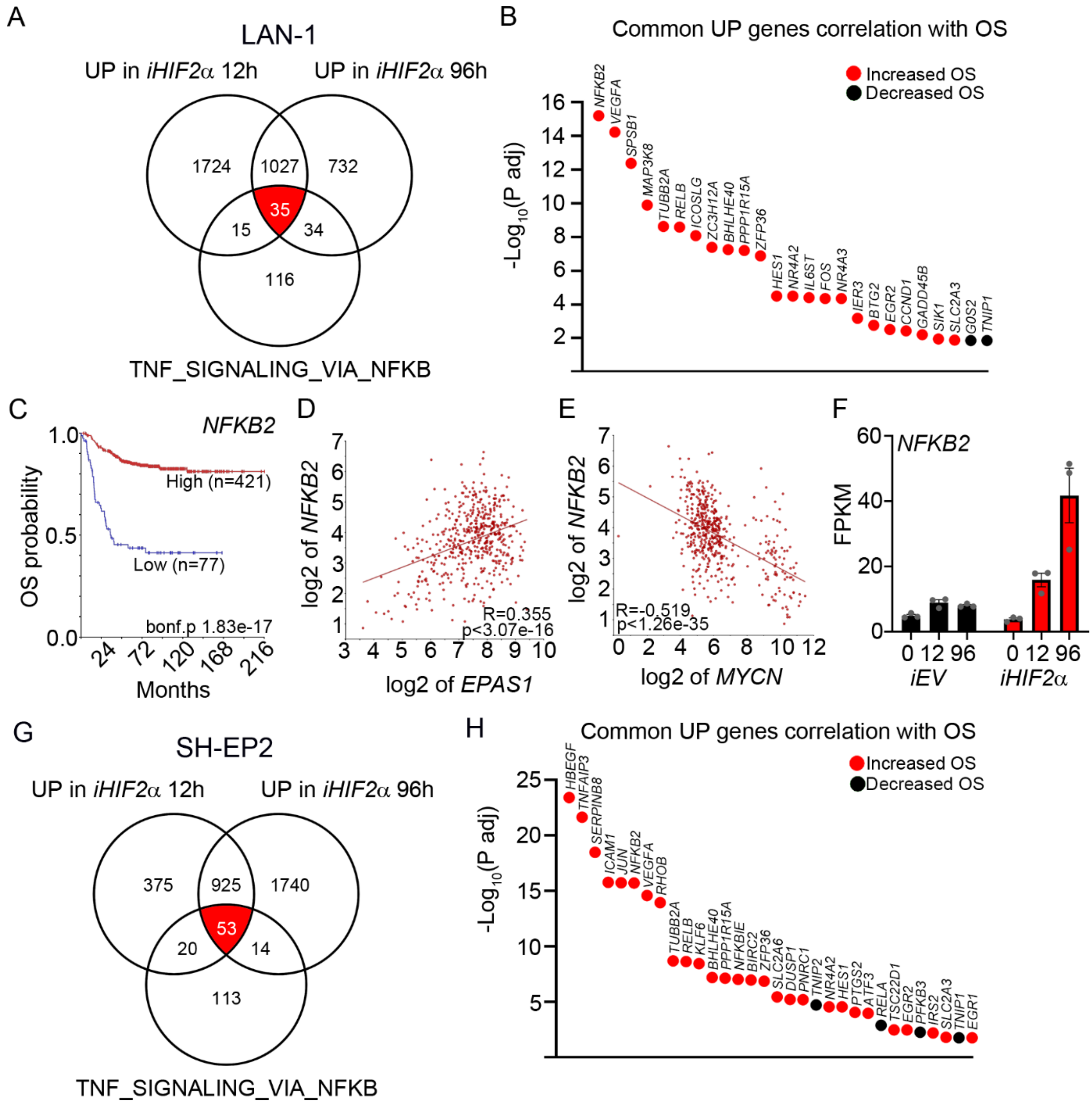

Supplementary Figure S10. Genes in the "TNFA signaling via NFKB" category which overlap with genes upregulated upon *iHIF2α* at 12 and 96 hours are enriched for factors predicting increased overall survival.

(A) Overlap between genes significantly upregulated at 12 and 96 hours after *iHIF2α* induction and genes in the "TNFA signaling via NFKB".

(B) Among the genes from the overlap in (A), 23 are significantly correlated with increased overall survival (red) whereas two are associated with decreased overall survival (black).

(C) *NFKB2* expression is significantly correlated with increased overall survival.

(D) *EPAS1* expression is positively correlated with *NFKB2* expression.

(E) Expression levels of *NFKB2* after 12- and 96-hour induction of *iHIF2α*

Supplementary Datasets

Dataset S1. Significance values between expression of indicated genes in clusters as defined in NBAtlas. Related to Fig. S2.

Dataset S2. Genes expressed at significantly higher levels (p adj.<0.05) in *iHIF2*α expressing LAN-1 cells compared to *iEV* expressing LAN-1 cells 12 hours after doxycycline induction.

Dataset S3. Genes expressed at significantly lower levels (p adj.<0.05) in *iHIF2*α expressing LAN-1 cells compared to *iEV* expressing LAN-1 cells 12 hours after doxycycline induction.

Dataset S4. Genes expressed at significantly higher levels (p adj.<0.05) in *iHIF2*α expressing LAN-1 cells compared to *iEV* expressing LAN-1 cells 96 hours after doxycycline induction.

Dataset S5. Genes expressed at significantly lower levels (p adj.<0.05) in *iHIF2*α expressing LAN-1 cells compared to *iEV* expressing LAN-1 cells 96 hours after doxycycline induction.

Dataset S6. Genes expressed at significantly higher levels (p adj.<0.05) in *iHIF2*α expressing SH-EP2 cells compared to *iEV* expressing LAN-1 cells 12 hours after doxycycline induction.

Dataset S7. Genes expressed at significantly lower levels (p adj.<0.05) in *iHIF2*α expressing SH-EP2 cells compared to *iEV* expressing LAN-1 cells 12 hours after doxycycline induction.

Dataset S8. Genes expressed at significantly higher levels (p adj.<0.05) in *iHIF2*α expressing SH-EP2 cells compared to *iEV* expressing LAN-1 cells 96 hours after doxycycline induction.

Dataset S9. Genes expressed at significantly lower levels (p adj.<0.05) in *iHIF2*α expressing SH-EP2 cells compared to *iEV* expressing LAN-1 cells 96 hours after doxycycline induction.

Supplementary Methods

EdU proliferation assay

The assay was performed according to the Kit protocol (C10637, ThermoFisher Scientific) Briefly, 20µM EdU was added to each experimental point for the last one hour of the total treatment duration (on 23rd hour for 24 hours of Dox treatment). Following an hour of incubation, cells were fixed with 3.5% PFA for 15 minutes followed by permeabilization in 0.5% TritonX-100 in 1X PBS for 20 minutes. Meanwhile, a mixture of reaction buffer, Copper protectant, Alexa Fluor 488, EdU buffer additive was prepared and added to each point after 20 minutes of permeabilization. Upon completion of 30 minutes, reaction mixture was removed. Cells were then washed twice and subjected to general protocol for counterstaining with desired antibodies (HIF2α/HA/MAP2).

PI staining and cell cycle analysis

For SK-N-BE(2) cells in Fig. S5Q: After 24 hours of Dox treatment, cells were dispersed into single cells with 1X PBS-0,5mM and collected on ice. Cells were then washed with ice cold 1X PBS for three times followed by fixation and immobilization in ice cold 70% ethanol for one hour. After one hour, fixed cells were washed twice with ice cold 1X PBS and then subsequently incubated with a propidium iodide (PI) staining solution containing 100µg/mL RNase A, 0.1 % Triton-X 100 and 10µg/mL PI for 30 minutes. Stained cells were washed with 1X PBS twice and subjected to flow cytometer (Accuri C6+, BD Biosciences). All experimental samples were prepared in triplicate. Data were collected from 10000 gated cells and analysed further.

For LAN-1 cells in Fig. 2N and SH-EP2 cells in Fig. 5G: Following the respective treatments, cells were harvested by trypsinization and fixed with pre-chilled 70% ethanol (EtOH) at 4°C for 1 hour. After fixation, cells were washed with phosphate-buffered saline (PBS) and incubated on ice for 15 minutes in PBS containing 0.25% Triton X-100 to permeabilize the membranes. Cell pellets were then resuspended in PBS containing 10 µg/mL RNase A and 20 µg/mL propidium iodide (PI) and incubated

at room temperature in the dark for 30 minutes. Cell cycle analysis was performed using flow cytometry (BD FACSCanto II). All experimental samples were prepared in triplicate. Flow cytometry data were analyzed using FlowJo software.

Table 1. List of antibodies

**Target**	**Cat.No.**	**Supplier**	**Application**	**Host**
**b-ACTIN**	A5441	Sigma	WB	Mouse
**GAPDH**	ab22555	abcam	WB	Rabbit
**HA**	sc-7392	Santa Cruz	WB, ICC	Mouse
**HA**	3724S	Cell Signaling	WB, ICC, IHC	Rabbit
**FLAG**	F1084	Sigma	WB	Mouse
**HIF2α**	7096	Cell Signaling	WB	Rabbit
**Ki-67**	ab16667	Abcam	WB, IHC, ICC	Rabbit
**Ki-67**	14-5698-82	ThermoFisher	IHC	Rat
**MYCN**	sc-791	Santa Cruz	WB	Rabbit
**MYCN**	sc-53993	Santa Cruz	WB, ICC	Mouse
**MYC**	sc-40	Santa Cruz	WB	Mouse
**DBH**	8586	Cell Signaling	WB,	Rabbit
**DDC**	sc-293287	Santa Cruz	WB, ICC	Mouse
**TH**	P60101-150	Pel-Freez Biologicals	IHC, ICC	Sheep
**TH**	P40101-150	Pel-Freez Biologicals	WB, IHC, ICC	Rabbit
**ASCL1**	sc-390794	Santa Cruz	WB	Mouse
**PHOX2B**	sc-376997	Santa Cruz	WB	Mouse

**HIF1α**	NB100-479	Novus	WB	Rabbit
**TUJ1**	MMS-435P-250	Biolegend	ICC	Mouse
**TUJ1**	PRB-435P-100	Biolegend	ICC	Rabbit
**MAP2**	ab5622	Sigma	ICC	Rabbit
**GFP**	A-11120	ThermoFisher	ICC	Mouse
**Anti-mouse IgG, HRP-linked**	7076s	Cell Signaling	WB	Horse
**Anti-rabbit IgG, HRP-linked**	7074s	Cell Signaling	WB	Horse
**Donkey anti-Mouse IgG (H+L) Alexa Fluor™ 488**	A-21202	ThermoFisher	ICC, IHC	Donkey
**Donkey anti-Mouse IgG (H+L) Alexa Fluor™ 555**	A-31570	ThermoFisher	ICC, IHC	Donkey
**Donkey anti-Mouse IgG (H+L) Alexa Fluor™ 647**	A-31571	ThermoFisher	ICC, IHC	Donkey
**Donkey anti-Rabbit IgG**	A-21206	ThermoFisher	ICC, IHC	Donkey

**(H+L) Alexa Fluor™ 488**				
**Donkey anti-Rabbit IgG (H+L) Alexa Fluor™ 555**	A-31572	ThermoFisher	ICC, IHC	Donkey
**Donkey anti-Rabbit IgG (H+L) Alexa Fluor™ 647**	A-31573	ThermoFisher	ICC, IHC	Donkey
**Donkey anti-Rat IgG (H+L) Alexa Fluor™ 488**	A-21208	ThermoFisher	ICC, IHC	Donkey
**Donkey anti-Sheep IgG (H+L) Alexa Fluor™ 488**	A-11015	ThermoFisher	ICC, IHC	Donkey
**Donkey anti-Sheep IgG (H+L) Alexa Fluor™ 555**	A-21436	ThermoFisher	ICC, IHC	Donkey

RNAscope

Frozen neuroblastoma (NB) patient tissue was embedded in optimal cutting temperature compound (O.C.T.; Sakura Finetek) and cryosectioned at 10 μm on a Cryotome maintained at the manufacturer-recommended temperature. Sections were stored at −80 °C until processing. Multiplex fluorescent RNA in situ hybridization was carried out with the RNAscope Multiplex Fluorescent Detection Kit v2 (Advanced Cell Diagnostics) according to the supplied protocol. Human-specific probes targeting EPAS1 (ACD #410591-C2), TH (ACD #441651-C3), and ENG (ACD #484111-C1) were hybridized. Fluorescent signals were captured on an LSM 700 confocal laser-scanning microscope (Zeiss).
